# A Review of Integrated Systems Based on Perovskite Solar Cells and Energy Storage Units: Fundamental, Progresses, Challenges, and Perspectives

**DOI:** 10.1002/advs.202100552

**Published:** 2021-05-19

**Authors:** Xuefeng Zhang, Wei‐Li Song, Jiguo Tu, Jingxiu Wang, Mingyong Wang, Shuqiang Jiao

**Affiliations:** ^1^ State Key Laboratory of Advanced Metallurgy University of Science and Technology Beijing Beijing 100083 P. R. China; ^2^ Institute of Advanced Structure Technology Beijing Institute of Technology Beijing 100081 P. R. China

**Keywords:** efficiency, integration systems, perovskite solar cells, rechargeable batteries

## Abstract

With the remarkable progress of photovoltaic technology, next‐generation perovskite solar cells (PSCs) have drawn significant attention from both industry and academic community due to sustainable energy production. The single‐junction‐cell power conversion efficiency (PCE) of PSCs to date has reached up to 25.2%, which is competitive to that of commercial silicon‐based solar cells. Currently, solar cells are considered as the individual devices for energy conversion, while a series connection with an energy storage device would largely undermine the energy utilization efficiency and peak power output of the entire system. For substantially addressing such critical issue, advanced technology based on photovoltaic energy conversion–storage integration appears as a promising strategy to achieve the goal. However, there are still great challenges in integrating and engineering between energy harvesting and storage devices. In this review, the state‐of‐the‐art of representative integrated energy conversion–storage systems is initially summarized. The key parameters including configuration design and integration strategies are subsequently analyzed. According to recent progress, the efforts toward addressing the current challenges and critical issues are highlighted, with expectation of achieving practical integrated energy conversion–storage systems in the future.

## Graphical abstract

The integrated energy conversion–storage systems (ECSISs) based on combining photovoltaic solar cells and energy storage units are promising self‐powered devices, which would achieve continuous power supplies for 24 h. Particularly, this novel combination technology can provide new markets for extending the energy application from battery‐based electric vehicles and smart grids.

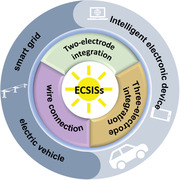

## 1 Introduction

Due to the resource shortage of fossil fuels and environmental crisis caused by CO_2_ and other greenhouse gases emissions, the global demands for green sustainable energy resources have attracted increasing attention. Currently the oil resources can only support exploitation for about 50 years.^[^ ^1^ ^]^ According to the statistics, the global energy consumption is estimated to reach approximately 27 (TW) by 2040.^[^ ^2^ ^]^ Although improving energy efficiency and conservation is beneficial to alleviate the energy crisis, investment of sustainable clean energy resources is the substantial to implementation and update of the global energy strategy.

As a typical form of solar system, sunlight is an essential renewable energy resource. In recent years, solar energy plays a critical role in water splitting, organic contaminant decomposition, energy conversion, and storage.^[^ ^3^ ^]^ Additionally, the development of solar cell with capabilities of converting solar energy to electricity is a direct strategy for utilizing energy resource. In the past several decades, great efforts have been paid to promote the stability and safety of solar cells. At present, silicon‐based solar cells, involving monocrystalline silicon,^[^ ^4^, ^5^ ^]^ polycrystalline silicon,^[^ ^5^, ^6^ ^]^ and amorphous silicon thin film solar cells, are the dominant products in the market.^[^ ^7^ ^]^ Nevertheless, there are still many limiting factors, including high energy consumption, large expenses, limited bandgap adjustability, and even the theoretical power conversion efficiency (PCE) of a single‐junction cell is only 29.1–29.4%.^[^ ^8^ ^]^ In addition, the thin‐film solar cells with alloys or compounds were also extensively investigated in early study, such as Sb_2_Se_3_,^[^ ^9^ ^]^ CdTe,^[^ ^10^ ^]^ GaAs,^[^ ^11^ ^]^ CuInSe_2_, etc.,^[^ ^12^ ^]^ while practical application of these materials is still restricted due to their toxicity. Moreover, dye‐sensitized solar cells (DSSCs) and organic compound solar cells show lower PCE (<14.3% for the former and 16% for the latter) than Si‐based solar cells.^[^ ^13^, ^14^ ^]^ Thus, the next generation solar cells are required to be low‐cost, high‐efficiency, and environmentally benign. In recent years, perovskite solar cells (PSCs) have attracted great attention as a promising candidate due to the unique advantages. i) Different from DSSCs, solid electrolytes could be employed into PSCs, which effectively overcomes the challenges such as electrolyte volatilization, electrolyte leakage, and encapsulation difficulty. ii) The Shockely–Queisser (S–Q) theoretical prediction suggests that the PCE is as high as 30%.^[^ ^15^ ^]^ iii) The raw materials of PSCs are mostly liquid, which can be easily used to prepare large‐area, low‐cost, and environment‐friendly flexible cells and devices.^[^ ^16^ ^]^ iv) In PSCs, unique features are desirable, including flexible bandgap, high optical absorption coefficient, low exciton bind energy, equilibrium carrier mobility, and long photocarrier life.^[^ ^17^ ^]^

However, solar cells possess the abilities of converting sunlight into electricity, while the converted energy cannot be harvested or stored. Therefore, it is necessary to exploit high‐performance integrated energy conversion–storage systems to meet the high demand for uninterrupted energy resource. Such integrated system is defined as the combination of the energy conversion unit (solar cells) and storage unit (metal‐ion batteries and supercapacitors). Noticeably, the overall photoelectric conversion and storage efficiency is an important indicator, which is substantially related to the PCE of solar cells. Although the integrated power packs upon tandem DSSCs and energy storage devices (Li‐ion batteries, LIBs for short, and supercapacitors) have been well fabricated, the overall photoelectric conversion and storage efficiency are still unsatisfied due to the low PCE of the DSSC module.^[^ ^18^ ^]^ Therefore, PSCs with higher efficiency exhibit greater potential as energy conversion unit in the integrated system.

For well understanding current state and challenges of the integrated energy conversion–storage systems, in this review, the integration of PSCs and energy storage devices is discussed and evaluated. First, the fundamental of PSCs is summarized, which includes operation principles, key parameters, critical problems, and challenges. As the critical support, design and fabrication techniques are specifically analyzed in the realization of integrated energy conversion–storage systems based on PSCs. In addition, the currently reported conversion systems will be discussed with consideration of various energy storage units, such as PSCs–LIBs, PSCs–supercapacitors, and PSCs with other types of energy storage devices. Finally, the challenges and future perspectives of conversion systems are highlighted, with expectation of paving the pathway from laboratory to industry.

## 2 Integrated Energy Conversion–Storage Systems Based on PSCs

### 2.1 Developing Demands

Recently, smart consumer electronics, electric vehicles, and smart grids are widely applied in the market, and rechargeable batteries are considered as the key energy storage devices in these products. In smart electronic devices, the capacity and energy density of batteries is still limited. Currently, the electrical power for rechargeable batteries mainly comes from the conversion of fossil energy. On the contrary, electrical power from solar energy conversion brings a green sustainable approach for battery charge due to the high‐power density of 100 mW cm^−2^ from the outdoor sunlight. On the other hand, electric vehicles assembled with power LIBs have activated a booming market, while the generated grid electricity is mainly from the coals and fossil fuels with emission of unexpected CO_2_. In addition, the requirement of wide distribution of charging stations is still a challenge to fulfill the large‐scale charging demand. Therefore, the power generation and distributed charging stations are the essential parts in the market of electric vehicles.

For addressing such energy bottleneck, efforts have been largely paid to the strategies of generating electricity from renewable energy sources with reduced harmful climate impact. In a typical progress, electric grid has been electrically connected to the photovoltaic power station. For further extending the utilization from daytime to nighttime, development of integrated energy conversion–storage systems could be considered as a potential strategy to connect to the grid. In this system, the generated electricity in the daytime could be stored into the integrated rechargeable batteries or supercapacitors, while the stored energy could be output in the nighttime to achieve a sustainable energy utilization.

### 2.2 Technique Requirement

In the applications, PCE is known to be one of the critical criteria and substantial improvement has been made in the PSCs. In principle, higher PCE implies the increased photon energy that is converted into electricity for charging energy storage device. PSC‐based integrated energy conversion–storage systems are attractive in the potential development, due to their unique advantages, such as all‐solid‐state form, high open circuit voltage, structural compliance, flexibility, active contact area shared with the coupled unit, and high theoretical PCE.

For rationally promoting the practical PCE, it is highly important to understand the fundamental of the integrated system. To achieve the goals, critical requirements, including high compatibility, ideal compactness (small integration volume), and lightweight portability, are the challenges for applications. Another key target is high energy storage efficiency, which can be calculated by the following equation^[^ ^19^ ^]^

(1) $$\eta_{3}=\eta_{2}/\eta_{1}\;\times \;100\%$$

where *η*_1_ is PCE and *η*_2_ is energy‐conversion and storage efficiency for the entire integrated system, which could be described as

(2) $$\eta_{2}=E_{d}/\left(P\times S\times t\right)\times 100\%$$

where *E*_d_, *P*, *S*, and *t* are discharge energy of energy storage device (mWh), light power density (mW cm^−2^), effective area of PSCs in series (cm^−2^), and photocharge time (h), respectively.

**Figure 1:**
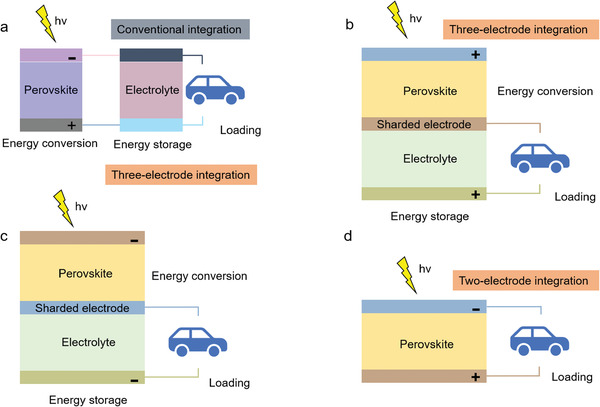
Circuit representation of PSCs–battery/supercapacitor systems. a) Conventional discrete charging. b) Three‐electrode configuration with common anode. c) Three‐electrode configuration with common cathode. d) Two‐electrode configuration.

Generally, there are two main routes in the integration of PSCs. i) The first type is the mechanical connection of two or more individual devices by a wire or stacking (**Figure** **1**a), by which the unit can operate simultaneously or independently. ii) Another configuration is a three‐electrode (Figure 1b,c) or two‐electrode integration (Figure 1d). In a three‐electrode configuration, one of the electrodes is shared by PSCs and energy storage units, which act as the positive electrode or negative electrode. In the two‐electrode configuration, on the other hand, the positive electrode has an integrated function, i.e., both photoconversion and energy storage.

### 2.3 Fundamental of Perovskite Solar Cells

#### 2.3.1 Configuration and Operation Principles

**Figure 2:**
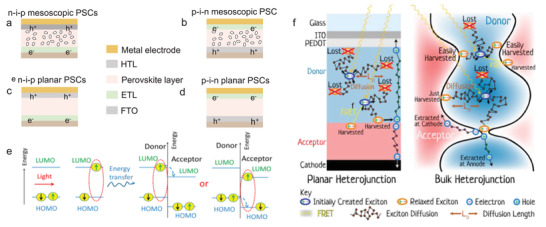
The configuration and operation principles of PSCs. a) n–i–p mesoscopic. b) p–i–n mesoscopic. c) n–i–p planar. d) Device structures of (c). e,f) The two different structures of heterojunction involve the generation of excitons, diffusion, and dissociation. Reproduced with permission.^[^ ^25^ ^]^ Copyright 2017, American Chemical Society.

PSCs are simply divided into organic–inorganic hybrids and all inorganic PSCs, which possess an all‐solid‐state light‐absorbing perovskite. In a typical configuration, PSCs consist of substrate materials (indium tin oxide(ITO) or fluorine‐doped tin oxide (FTO)) , electron transport layer (ETL) (TiO_2_, SnO_2_, and ZnO),^[^ ^20^ ^]^ perovskite absorption layer, hole transport layer (HTL), and metal electrode (**Figure** **2**a–d). PSCs exhibit regular (n–i–p) and inverted (p–i–n) structures, which depend on the transport (electron/hole) material that is presented on the exposing surface for interacting with the incident light. According to the design principles, there are four types of sandwiched PSC structures: n–i–p mesoscopic PSCs (Figure 2a), p–i–n mesoscopic PSCs (Figure 2b), n–i–p planar PSCs (Figure 2c), and p–i–n planar PSCs (Figure 2d). To date, both mesoporous and planar structures of PSCs have exhibited high performance and stability, while their stability is still under debate.^[^ ^21^ ^]^ The planar architecture is an evolution configuration of the mesoscopic structure, where the perovskite light‐harvesting layer is sandwiched between the ETL and HTL. With the same materials and approaches, a planar n–i–p PSC exhibits higher value of *V*_oc_ and *J*_sc_ in comparison with mesoscopic ones. However, more severe *J*–*V* hysteresis would be obtained probably owing to the incompatibility of p‐type materials, voltage scan direction, scan rate and range.^[^ ^22^ ^]^ In an inverted (p–i–n) structure, typical feature includes low‐temperature processing, negligible hysteresis behavior and optically attractive for (two/four‐terminal) tandem applications.^[^ ^23^ ^]^ However, further attempts should be made to understand the difference between mesoscopic inverted structure and planar inverted structure.

In the operation, the perovskite layer of PSCs first absorbs photons (*E*_hv_ > *E*_g_) to generate electron–hole pairs.^[^ ^2^ ^]^ Upon absorption of a photon, the electron could be excited from the semiconductor valence band to the conduction band, leaving a hole in the original position.^[^ ^24^ ^]^ Since the dielectric constants in perovskite materials (organic and inorganic materials) are different, free carriers or excitons could also be generated.^[^ ^25^, ^26^ ^]^ Subsequently, these uncombined electrons and holes (or dissociated excitons) are collected by the ETL and HTL, respectively. Specifically, the electrons transport from the perovskite layer to the ETL, and then are collected by the ITO. On the other hand, the holes transport from the perovskite layer to the HTL, and are further collected by the metal electrode. Finally, the photovoltage and photocurrent are generated by the electrically connecting the ITO and metal electrode.

Generally, the essential difference between inorganic semiconductors and excitons of organic semiconductors refers to the generation of photons excitation, while they are difficult to transfer to the electron and hole transport layers for generating photocurrent and photovoltage. Excitons are composed of Coulomb‐bound electron–hole pairs, which are usually defined as singlet excitons with a binding energy between 0.1 and 0.4 eV.^[^ ^27^ ^]^ In order to utilize these excitons in an external circuit, they must be first dissociated into free electron–hole pairs by using a heterojunction (p–n) assembled with electron donor and acceptor materials.^[^ ^28^ ^]^ Upon absorbing a photon, one of the two electrons with opposite spin directions in the highest occupied molecular orbital (HOMO) will be driven to the lowest unoccupied molecular orbital (LUMO), due to the spin conservation (Figure 2e). Meanwhile, the HOMO and LUMO energies of the acceptors are lower than those of the donors, resulting in the dissociation of electrons and holes driven by the energy offset.^[^ ^29^ ^]^ Generally, there are two different structures of heterojunction to harvest as much light as possible, i.e., planar heterojunction (Figure 2f, left) and bulk heterojunction (Figure 2f, right). In particular, the bulk heterojunction is defined as a uniform mixture of the donor and acceptor materials, in which excitons can spontaneously dissociate into electron–hole pairs.^[^ ^30^ ^]^ Primarily, the excitons generated from donors are unable to be dissociated until they reach the donor–acceptor interfaces through the different diffusion paths, which indicates that excitons are dissociated at the interface. Furthermore, the thickness‐scale distribution of the heterojunction is important for capturing excitons. In principle, a large thickness distribution can result in the loss of excitons while a small thickness distribution is beneficial to harvest excitons to achieve dissociation. Regarding the harvesting and dissociation of excitons in different heterojunctions, Samuel et al.^[25]^ have presented a deeper comprehension in the previous studies.^[^ ^2^, ^26^, ^31^ ^]^

#### 2.3.2 Key Parameters

For over decades, considerable efforts on PSCs have been made to achieve the PCE values to approach the S–Q limit. In the past 10 years, PSCs have achieved remarkable increased PCE from 3.8% (data recorded in 2009) to 25.2% (data recorded in 2019).^[^ ^32^, ^33^ ^]^ In the photoelectric conversion process, solar energy is directly converted into electricity by photovoltaic effect. The PCE (*η*_1_) is determined by several key parameters, such as *V*_oc_, *FF*, and *J*_sc_, which can be calculated by the following equation^[^ ^34^ ^]^

(3) $$\eta_{1}=V_{\mathrm{oc}}\times \mathrm{FF}\times J_{\mathrm{sc}}/P\times 100\%$$

where *FF*, *V*_oc_, *J*_sc_, and *P* are the fill factor, open‐circuit voltage (V), short‐circuit current density (mA cm^−2^), and incident light power density (mW cm^−2^), respectively. Therefore, maximizing these three key parameters is necessary to improve the photovoltaic performance. Note that these parameters are interrelated, and the corresponding values could be also impacted by other physical properties.

Generally, *V*_oc_ originates from the splitting of quasi‐Fermi energy levels for electrons and holes, which is activated by radiation of light^[^ ^35^ ^]^

(4) $$V_{\mathrm{oc}}=\left(E_{\mathrm{Fn}}-E_{\mathrm{Fp}}\right)$$

where *E*_Fn_ and *E*_Fp_ represents the electron and hole quasi‐Fermi levels, respectively. *V*_oc_ is significantly controlled by light absorption and carrier recombination. The recombination includes nonradiative recombination and radiative recombination. In the radiative recombination of PSCs, the upper limit for *V*_oc_ is usually determined as 1.32–1.34 V. On the other hand, the *V*_oc_ for nonradiative value is much lower than 1.1 V.^[^ ^36^ ^]^ Practically, several studies have demonstrated that the recombination of electron–hole pairs is mainly attributed to nonradiative recombination.^[^ ^37^ ^]^ Hence, it is an effective way to increase *V*_oc_ by eliminating nonradiative recombination.

Additionally, another important parameter is *FF*, which can be calculated by following equation^[^ ^38^ ^]^

(5) $$\mathrm{FF}=\left(V_{\mathrm{mp}}/V_{\mathrm{oc}}\right)\times \left(J_{\mathrm{mp}}/J_{\mathrm{sc}}\right)$$

where *V*_mp_, *J*_mp_, *V*_oc_, and *J*_sc_ are the maximum power point voltage, maximum power point current, open‐circuit voltage, and short‐circuit current density, respectively. Simply, the value of *FF* depends on the ratio of the transmission rate to the recombination rate of the device during operation. According to S–Q limit theory, however, it depends on the maximum power point voltage. Actually, series resistance (*R*_s_) and shunt resistance (*R*_sh_) are non‐negligible limiting factors in realistic device system, which can impact the value of *FF* and *V*_oc_.^[^ ^39^ ^]^ Generally, *FF* largely depends on the *R*_s_ and *R*_sh_, and both high *R*_s_ and low *R*_sh_ decrease the value of *FF*, i.e., higher *R*_sh_ can improve *FF* and electron mobility.^[^ ^40^ ^]^ Although high *FF* is necessary in high‐performance PSCs, it is difficult to investigate the effects of FF in a targeted manner. In a specific experiment, it is still a challenge to observe the changes of FF by varying one parameter while the other parameters remain constant.

High short‐circuit current density (*J*_sc_) is a critical parameter to achieve the theoretical limit of PCE, and *J*_sc_ is affected by light reflection losses, trap density, and interface control (electron transport layer and light absorbing layer). However, most experimental values of PSCs reach only 80% of its maximum *J*_sc_, in comparison with 90% achieved in the solar cells using Si and GaAs.^[^ ^41^ ^]^ Generally, the quantum efficiency is defined as the ratio of photon numbers collected on the absorber layer to the total ones on the solar cells. The photons are absorbed by the absorber layer, which would generate electrons and holes to transfer to their corresponding transport layer. This behavior could in turn contribute to the short‐circuit current. Therefore, the efficient photon management, trap density, and interface control (high conductivity and matching configuration) are required to be considered for maximizing the *J*_sc_.^[^ ^42^, ^43^ ^]^ For instance, Qarony et al. used nonresonant metal‐oxide metasurfaces to demonstrate the potential improvement in short‐circuit current density.^[^ ^42^ ^]^

#### 2.3.3 Critical Problems and Challenges

**Figure 3:**
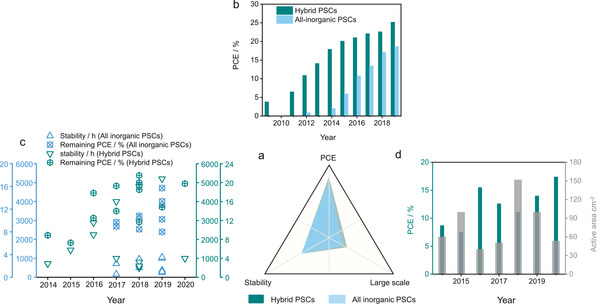
a–d) The challenges and state‐of‐the‐art of PSCs; the references are given in Tables 1 and 2.

Although the photovoltaic technology upon PSCs has achieved rapid development in recent years, critical problems and challenges have largely hindered the commercialization process (**Figure** **3**a and **Table** **1**). In the PCE, the value is close to those of Si‐based commercialized solar cells, while it still needs to be improved according to S–Q limit as aforementioned. Theoretically, maximizing *V*_oc_, *FF*, and *J*_sc_ via appropriate improvement strategies would promote PCE. Therefore, the entire structure configuration of PSCs is also required to possess high compatibility, such as the light‐absorbing layer, electron and hole transport layer. As shown in Figure 3b, the annual top PCEs of hybrid and all inorganic PSCs are listed.^[^ ^32^, ^33^, ^44^, ^45^, ^46^, ^47^, ^48^ ^]^ Additionally, stability is a necessary prerequisite for the long‐term operation in the PSCs. Although the stability of some PSCs devices has achieved a stable operation more than 30 days under a specific environment, there is still a huge gap in comparison with the commercial photovoltaic devices (such as stability and cost). Currently, the major challenges of perovskite materials refer to the many aspects, including crystal structure stability, environmental factors (such as temperature, O_2_, and H_2_O), and phase purity. There features would promote the processing requirements for achieving high‐quality perovskite films. Therefore, PSCs should operate efficiently under the environment with high oxygen concentration, high atmospheric humidity, or high temperature (Figure 3c). Compared with the stability and environmental factors, a more severe problem is the scalable processing for realizing large‐area manufacturing (Figure 3d). In detail, the main challenge of commercialization is inevitably efficiency loss, which is caused by the inferior quality of the film (e.g., pinhole, crack, and defect) during the scalable fabrication of perovskite films. Currently, perovskite films mainly rely on three preparation methods, such as single‐step solution deposition, two‐step solution deposition and vapor‐assisted solution deposition. Among these methods, vapor‐assisted solution deposition has been evidenced to be the most effective. Although lead (Pb) is abundant in the earth crust and lead‐based PSCs possess impressive potential PCE, it is necessary to investigate lead‐free PSCs from a safety concern. Therefore, the potential crisis of lead leakage should be substantially avoided. In the integrated energy conversion–storage systems, the overall stability, energy density, safety, and long‐term operation are highly dependent on PSCs. Therefore, considerable improvement on the PSCs is also an important factor for achieving high‐performance practical integrated energy conversion–storage systems.

**Table 1:** The progress in the stability of perovskite solar cells

Device configuration	PCE [%]	Stability conditions	Remaining PCE [%]	Year^[Ref.]^
FTO/c‐TiO_2_/CsPb_0.96_Bi_0.04_I_3_/CuI/Au	13.21	Unencapsulated for 168 h under ambient conditions	8.98	2017^[^ ^119^ ^]^
ITO/TiO_2_/CsPbI_2_Br/P_3_HT/Au	12.02	Unencapsulated for 960 h in a dry glovebox	10.8	2018^[^ ^120^ ^]^
FTO/TiO2/graphene Ds/CsPbBr_3_/C	9.72	Unencapsulated in RH 90% 25 °C for 130 days	8.46	2018^[^ ^121^ ^]^
ITO/TiO_2_/CsPbI_2_Br/PTAA/Au	14.86	Unencapsulated under continuous 1 sun light soaking at 85 °C for 1000 h	13.37	2019^[^ ^122^ ^]^
ITO/PTAA/CsPbI_2.94_Cl_0.06_/PCBM/C60/BCP/Al	11.4	Unencapsulated in air for over 30 days	9.69	2017^[^ ^123^ ^]^
N‐GQD/FTO/TiO_2_/*γ*‐CsPbI_3_/PTAA/Au	16.02	10 days under 50% RH in N_2_ atmosphere	15.7	2019^[^ ^124^ ^]^
FTO/c‐TiO_2_/PTABr‐CsPbI_3_/spiro‐OMeTAD/Ag	17.06	Unencapsulated in N_2_ glovebox (500 h of continuous white light LED illumination)	16.3	2018^[^ ^125^ ^]^
FTO/cTiO_2_/CsPbI_2_Br/carbon	10.21	Long‐term stability with no obvious efficiency degradation under ambient atmosphere at 15–30% RH at room temperature for 44 days	10.21	2019^[^ ^126^ ^]^
FTO/c‐TiO_2_/m‐TiO_2_/CsPb_1_ *_x_* Eu*_x_* I_2_Br(0 ≤ *x* ≤ 1)/spiro‐OMeTAD/Au	13.71	The devices retain 93% of the initial efficiency after 370 h under 100 mW cm^−2^ continuous white light illumination	12.75	2019^[^ ^127^ ^]^
ITO/TiO_2_/Cs_1.2_PbI_2_Br_1.2_/P3HT/Au	10	Unencapsulated at 70 °C in inert atmosphere for 300 h	8	2019^[^ ^128^ ^]^
FTO/TiO_2_/MAPbI_3−_ *_x_* Cl*_x_*/sputtered NiO*_x_*/Ni	7.28	>2 months stability		2015^[^ ^129^ ^]^
FTO/c‐TiO_2_/mp‐TiO_2_/(FAPbI_3_)*_x_* (MAPbBr_3_)_1−_ *_x_*/spiro‐MeOTAD/Au	18.7	3 months outdoor	17.76	2016^[^ ^130^ ^]^
FTO/PEDOT:PSS/(BA)_2_(MA)_3_Pb_4_I_13_/PCBM/Al	12.52	Under 1 sun 2250 h, 65% RH	12.52	2016^[^ ^131^ ^]^
	17.5	4000 h in air	14	2017^[^ ^132^ ^]^
FTO/bl‐TiO_2_/mp‐TiO_2_/CsFAMAPbI_3−_ *_x_* Br*_x_*/CuSCN/rGO/Au	20.4%	95% left after 1000 h under 60 °C	19.38	2017^[^ ^133^ ^]^
MAPb(I_1−_ *_x_* Br*_x_*)_3−_ *_y_* Cl*_y_*	11.1	80% for 720 h	8.88	2014^[^ ^134^ ^]^
FTO/NiO*_x_*/FA_1−_ *_x_* MA*_x_* PbI_3_/PCBM/TiOX/Ag	20.65	90% left after 500 h under 85 °C	18.5	2018^[^ ^135^ ^]^
FTO/BI‐TiO_2_/mp‐TiO_2_/FA‐perovskite/spiro‐OMeTAD/Au	20	800 h	20	2018^[^ ^136^ ^]^
FTO/c‐TiO_2_/mp‐TiO_2_/(FAPbI_3_)_0.85_(MAPbBr_3_)_0.15_/spiro‐OMeTAD/Au	14.6	Up to 80% PCE retained over 200 days (ambient atmosphere, 50% relative humidity, unencapsulated, stored in dark), <50% PCE retained	11.68	2018^[^ ^137^ ^]^
FTO/TiO_2_/perovskite/spiro‐MeOTAD/Au	21.52	100% PCE retained at 25 °C, 79% PCE retained at 50 °C (maximum power point, N_2_ atmosphere, 600 h)	21.52	2018^[^ ^138^ ^]^
FTO/NiO/(FA_0.83_MA_0.17_)_0.95_Cs_0.05_Pb(I_0.9_Br_0.1_)_3_/PVBM/BCP/Au	19.38	1 sun 70–75 °C 5400 h	14.8	2019^[^ ^139^ ^]^
ITO/PTAA/perovskite/PC61BM/EEL/Ag	22.02	1000 h at 85 °C	19.8	2020^[^ ^140^ ^]^

#### 2.3.4 Large‐Scale Preparation and Low Toxicity of PSCs

Since 2012, the efficiency of PSCs has evidenced an amazing growth trend.^[^ ^32^ ^]^ Up to date, the certified efficiency has reached 25.2%,^[^ ^33^ ^]^ which has exceeded the traditional photovoltaic technology. However, the efficiency of large‐area devices is still low. In detail, the efficiency loss would occur during the scalable fabrication of perovskite films, which is mainly due to the quality degradation of the film as the fabrication area increases (e.g., pinhole, crack, and defect). The reliable high‐efficiency fabrication of high‐quality large‐area perovskite films should be the critical in upscaling commercialization of PSCs. Therefore, the fabrication method of the perovskite film is of great significance for realizing large‐scale preparation. In this section, we have reviewed several main preparation methods and evaluated their feasibility in large‐scale fabrication, such as solution deposition (spin‐coating, blade coating, slot die coating, and spray coating), chemical vapor deposition (CVD), and hybrid chemical vapor deposition (HCVD).^[^ ^49^, ^50^, ^51^ ^]^

First, solution‐based scalable techniques have been widely employed in the preparation of high‐quality perovskite films due to its low‐cost and facile feature. In spin‐coating, numerous efforts on the lab‐scale small‐area and scale‐up‐area setups have been made, which suggests that it is common and available.^[^ ^52^, ^53^, ^54^ ^]^ The corresponding PCE reached as high as ≈13% and 17.1% with a reported active area size of 50.6 and 24.94 cm^2^, respectively.^[^ ^53^, ^55^ ^]^ However, it is a challenge to use this technique to deposit uniform perovskite films in a reproducible manner when the size is larger than 100 cm^2^.^[^ ^56^ ^]^ Therefore, the techniques of blade coating,^[^ ^57^, ^58^, ^59^ ^]^ slot die coating, and spray coating were reported in order to break the challenge.^[^ ^60^, ^61^, ^62^ ^]^ Among them, the cells obtained using blade coating^[^ ^61^ ^]^ (active area: 151.9 cm^2^, PCE: 11.1%) and slot die coating (active area: 57.2 cm^2^, PCE: 15%)^[^ ^57^ ^]^ delivered greater performance. When the films are in wet state, there would be a kinetic process of nucleation and crystal growth using blade coating, which is similar to that of spin‐coating. However, rapid removal of the solvent during film drying is the current challenge because the film quality would be significantly impacted using nonrotating coating technique. In the spin‐coating process, the substrate is rotated at a high speed in order to efficiently spread the extra solution out from the film owing to centrifugal force. For example, Lee et al.^[^ ^63^ ^]^ employed lead acetate (PbAc_2_) in the mother solution to form methylammonium acetate (MAAc) with purpose of controlling crystal growth during the drying process of the cast film. Other improvement strategies, such as introduction of surfactant (e.g., l‐*α*‐phosphatidylcholine)^[^ ^57^ ^]^ to form amine complex precursors (CH_3_NH_3_I·*m*CH_3_NH_2_ and PbI_2_·*n*CH_3_NH_2_), have been also proposed for fabricating MAPbI_3_ perovskite films.^[^ ^64^ ^]^

Vapor‐based deposition (e.g., CVD and HCVD) methods have also been demonstrated as a promising route to fabricate a large‐area, uniform, and pinhole‐free film. CVD process refers to the formation of a thin solid film on a substrate via a chemical reaction of vapor phase precursors at a precise temperature. Compared with solution coating processes, CVD deposition process exhibits unique advantages, such as easy formation of perovskite heterojunction structures,^[^ ^65^ ^]^ construction of full textured tandem‐structure solar cells,^[^ ^66^ ^]^ elimination of using harmful organic solvents, etc.^[^ ^67^ ^]^ Fan and co‐workers^[^ ^50^ ^]^ reported a facile one‐step CVD method to fabricate planar heterojunction PSCs (MAPbI_3_ and MAPbI_3−_ *_x_* Cl*_x_* perovskite) with a PCE up to 11.1%. Notably, all of the precursors in CVD process were solid state. Specifically, perovskite thin films were deposited onto a c‐TiO_2_‐coated FTO glass substrate by a one‐step method. The lead chloride (or lead iodide) and methylamine iodide were placed in the high temperature zone and the exact position of each source was determined according to their vaporization temperature. In the growth process, the substrates were placed in the left side low temperature zone. Due to the discrepancy in physical properties between different precursors, more stringent requirements (higher accuracy in carrier gas, pressure, and temperature) are necessary to fabricate high‐quality perovskite thin films. Qi and co‐workers proposed the HCVD technique in order to achieve a more flexible and simple preparation process.^[^ ^51^ ^]^ Typically, the perovskite film grown by the HCVD includes a two‐step process. The individual step can be optimized separately, which allows for controlling the process more accurately. Apart from this, vapor‐assisted solution process is also a promising approach, which combines solution process and vapor process. Such combination would effectively simplify the preparation process and technical requirements. In 2013,^[^ ^45^ ^]^ Snaith and co‐workers successfully fabricated MAPbI_3−_ *_x_* Cl*_x_* thin film by vapor‐assisted solution process, in which both ETL and HTL were obtained by spin‐coating process via the evaporation of organic and inorganic source.

In summary, the technologies aforementioned possess advantages and challenges, and they have been used for large‐scale preparation. The high quality of the perovskite film is the main technical barrier for both large‐area preparation and high PCE. Meanwhile, large‐area preparation is a necessary prerequisite for the commercial application of PSCs. Herein, we have collected prominent achievements in large‐scale preparation in recent years (**Table** **2**).

**Table 2:** Selected reports on large‐scale preparation of PSCs

Configuration of PSCs	Active area [cm^2^]	Deposition method	PCE [%]	Year^[Ref.]^
ITO/PEDOT:PSS/MAPbI_3_/PCBM/LiF/Al	60	Spin‐coating	8.7	2014^[^ ^54^ ^]^
ITO/PEDOT:PSS/MAPbI_3_/PCBM/Au	40	Spin‐coating	12.9	2015^[^ ^141^ ^]^
FTO/c‐TiO_2_/m‐TiO_2_/graphene/MAPbI_3_/spiro‐MeOTAD/Au	50.6	Spin‐coating	12.6	2017^[^ ^53^ ^]^
ITO/PEDOT:PSS/MAPbI_3−_ *_x_*_−_ *_y_* Br*_x_* Cl*_y_*/PCBM/Ca/Al	25.2	Spin‐coating	14.3	2016^[^ ^142^ ^]^
FTO/SnO_2_/K_0.03_Cs_0.05_(FA_0.85_MA_0.15_)_0.92_Pb(I_0.85_Br_0.15_)_3_/spiro‐OMeTAD/Au	53.64	Spin‐coating	17.4	2020^[^ ^143^ ^]^
FTO/SnO_2_/[CsPbI_3_]_0.05_[(FAPbI_3_)_0.85_(MAPbBr_3_)_0.15_]_0.95_/spiro‐MeTOAD/Au	25/100	Spin‐coating	15.3/14.0	2019^[^ ^144^ ^]^
Glass/FTO/c‐TiO_2_+G/m‐TiO_2_+G/perovskite/spiro‐OMeTAD/Au	70	Spin‐coating	14	2019^[^ ^145^ ^]^
FTO/c‐TiO_2_/m‐TiO_2_/MAPbI_3_/P_3_HT/Au	100	Blade coating	7.5	2015^[^ ^59^ ^]^
ITO/PTAA/MAPbI_3_/C_60_/BCP/Cu	33.0/57.2	Blade coating	15.3/14.6	2018^[^ ^57^ ^]^
ITO/PTAA/MAPbI_3_/C_60_/BCP/metal cathode	63.7	Blade coating	16.4	2019^[^ ^146^ ^]^
ITO/c‐TiO_2_/MAPbI_3−_ *_x_* Cl*_x_*/spiro‐MeOTAD/Au	151.9/142	Slot‐die coating	11.1/11.8	2018^[^ ^61^ ^]^
FTO/c‐TiO_2_/MAPbI_3−_ *_x_* Cl*_x_*/PTAA/Au	40	Spray coating	15.5	2016^[^ ^62^ ^]^
FTO/SnO_2_/(FA_0.85_MA_0.15_)_0.95_Pb(I_0.85_Br_0.15_)_3_/spiro‐MeOTAD/Au	53.6	Solvent‐bath process	13.9	2019^[^ ^147^ ^]^
FTO/SnO_2_/Cs*_x_* FA_1−_ *_x_* PbI_3−_ *_y_* Br*_y_*/spiro‐MeOTAD/Au	41.25	CVD	12.24	2018^[^ ^148^ ^]^
FTO/SnO_2_/C_60_/Cs_0.1_FA_0.9_PbI_3_/spiro‐MeOTAD/Au	82.6	HCVD	10.37	2019^[^ ^149^ ^]^

Although lead is abundant in the earth crust and lead‐based PSCs possess an impressive PCE, it is necessary to investigate lead‐free PSCs. Therefore, another development of PSCs is to delead in the technique, avoiding the threat of lead leakage to both humans and environment. The radius of Sn^2+^ is almost equal to that of Pb^2+^, which is considered as the substitute of Pb in terms of forming ASnI_3_ (A = MA, FA, etc.; X = Br, I, etc.) and CsSnI_3_ (1.3 eV). The Sn‐based PSCs possess a lower *E*_g_ than Pb‐based ones, which is closer to the ideal bandgap (1.34 eV) of the Shockley–Queisser limit for photovoltaic devices in comparison with that of lead‐based perovskites.^[^ ^68^ ^]^ Theoretically, tin‐based perovskites are very promising materials for PSCs. However, Sn^2+^ is extremely unstable because it can be easily oxidized to Sn^4+^ if exposing in the air or even in inert atmosphere. The self p‐doped Sn^4+^ (so‐called self‐doping effect) breaks the charge neutrality and forms high density of recombination centers in perovskite, which leads to PCE reduction.^[^ ^69^ ^]^ Additionally, “yellow” phase is also an unavoidable challenge. Therefore, it is a challenge to achieve Sn‐based PSCs with long‐term stability and desired PCE. In this part, we will mainly focus on the progress and discussion of inhibiting the oxidation of Sn^2+^ in Sn‐PSCs.

Actually, Noel et al.^[^ ^70^ ^]^ proposed lead‐free organic–inorganic hybrid PSCs (MASnI_3_, *E*_g_ = 1.23 eV) as early as 2014. They have determined the mobility of the as‐prepared films, with a value of 1.6 cm^2^ V^−1^ s^−1^ and a diffusion length of ≈30 nm. However, the main challenge is that the instability of Sn^2+^ oxidation state results in PCE of 6% and *V*_oc_ of 0.88 V. In the same year, MASnI_3−_ *_x_* Br*_x_* was employed as a light harvester into lead‐free PSCs. However, the as‐obtained PCE of 5.73% was still lower than that of the Pb‐based PSCs, which should be mainly attributed to the oxidation of Sn^2+^.^[^ ^71^ ^]^ Later, a 3D MASnI_3_ perovskite structure was designed by Kanatzidis and co‐workers,^[^ ^72^ ^]^ with a slightly improved PCE (6.63%) achieved. The oxidation of Sn^2+^ is a key challenge, leading to a higher carrier density and conductivity. Such results would short‐circuit the devices, which are needed to be solved urgently. Therefore, some groups attempted to employ SnF_2_, SnF_2_–pyrazine complex, Sn powder, FABr, (N_2_H_5_Cl), BAI, and EDAI_2_ as additives to suppress the oxidization of Sn^2+^, with purpose of reducing the hole density in the resulting films.^[^ ^48^, ^73^ ^]^ Another strategy is so‐called hollow perovskite using propylenediammonium c), trimethylenediammonium , and ethylenediammonium.^[^ ^74^ ^]^ Although the content of Sn^4+^ has been reduced, the PCE is still maintained at a low level below 7%, which is mainly attributed to the poor carrier transport properties. Similarly, Zhu et al.^[^ ^75^ ^]^ reported a Lewis acid‐base adduct strategy using trimethylamine (TMA) as the additional Lewis base in the tin halide solution to form SnY_2_–TMA complexes (Y = I^−^, F^−^), thus achieving a PCE of 7.09% in the inverted structure. In order to further suppress the oxidation of Sn^2+^, Tai et al.^[^ ^76^ ^]^ employed phenolsulfonic acid (PSA), 2‐aminophenol‐4‐sulfonic acid (APSA), and potassium salt of hydroquinone sulfonic acid (KHQSA) as antioxidant additives into the perovskite precursor solution along with excess SnCl_2_. Note that KHQSA contains two hydroxyls (—OH) groups, and have stronger interaction with Sn^2+^. As a result, higher antioxidant activity could be obtained. As expected, improved PCE (6.76%) and stability (80% efficiency maintenance over 500 h upon air exposure without encapsulation) was achieved due to the precise control of Sn^2+^ oxidation. Low‐dimensional perovskite (LDP) interlayer between ETL and perovskite was also reported to possess a PCE of 7.05%.^[^ ^77^ ^]^ Additionally, Ke et al.^[^ ^78^ ^]^ designed a novel tetrakistriphenylamine (TPE) small molecule as HTL to take place the conventional 2,2′,7,7′‐tetrakis(N,N‐di‐p‐methoxyphenyl‐amine) 9,9′‐spirobifluorene （spiro‐OMeTAD) and poly(bis(4‐phenyl)(2,4‐bimethylphenyl)amine （PTAA). Owing to the suitable band alignment and excellent hole extraction/collection properties, TPE HTL presented a PCE of 7.23% (*V*_oc_ = 0.459 V, *J*_sc_ = 22.54 mA cm^−2^; *FF* = 69.74%). Jokar et al.^[^ ^79^ ^]^ proposed a mixing strategy of a “A” site organic cation and additive engineering, and fabricated the GA*_x_* FA_1−_ *_x_*_−2_ *_y_* SnI_3_–*y*EDAI_2_ perovskite film, in which the EDAI_2_ effectively suppressed the oxidation of Sn^2+^ on the surface. An optimized performance (maximum PCE = 9.6%) was achieved at a precursor ratio (guanidinium iodine:formamidinium iodine (GAI:FAI))of 20:80 in a glove‐box environment for 2000 h. Notably, the *V*_oc_ and PCE from the above results are still lower than Pb‐based PSCs (especially *V*_oc_ generally below 1.0 V), which is mainly attributed to the transition of Sn^2+^ to Sn^4+^ and poor carrier transport capability.

Sn‐based all‐inorganic PSCs also become a significant developing direction for free‐lead PSCs due to the bandgap close to the S–Q limit. CsSnI_3_ is a unique phase‐transition material, which exhibits two polymorphs at room temperature. One has a 1D yellow double‐chain structure (Y‐CsSnI_3_) and the other has a 3D black perovskite structure (B‐*γ*‐CsSnI_3_ with low exciton binding energy of 10–20 meV). The black phase is a highly conductive p‐type direct semiconductor with a bandgap of 1.3 eV, which possesses a photoelectric response. In contrast, the yellow phase is an indirect semiconductor with a 2.55 eV bandgap.^[^ ^80^ ^]^ However, individual CsSnI_3_ is not effective, because it exhibits metallic conductivity and is prone to form intrinsic defects of both Sn vacancies and Sn^4+^ centers. In 2012, B‐*γ*‐CsSnI_3_‐based PSCs were first reported and a PCE of only 0.9% was achieved due to the oxidation of Sn^2+^.^[^ ^47^ ^]^ Thus, it is critical to control the inherent defect concentration to optimize the all‐inorganic Sn‐based PSCs. Later, Sn‐containing compounds, such as SnF_2_, SnCl_2_, and SnI_2_, were demonstrated, and they were beneficial to enhance the performance via decreasing the intrinsic defects density. For example, Mathews et al.^[^ ^48^ ^]^ demonstrated that the carrier density of CsSnI_3_ was observed to decrease with increasing SnF_2_. Thus, the addition of SnF_2_ would reduce the concentration of Sn vacancies. The devices with the configuration of FTO/compact TiO_2_/mesoporous TiO_2_/CsSnI_3_/HTL/Au exhibited PCE of 2.02%, *V*_oc_ of 0.16 V, *J*_sc_ of 22.7 mA cm^−2^, and *FF* of 37%. SnF_2_ can improve the stability, it also remains intact in the film owing to its chemical stability. Thus, the formation of Sn defects from such oxidization might be rarely prevented with addition of SnF_2_. Hatton and co‐workers used SnF_2_, SnCl_2_, and SnBr_2_ as additives in CsSnI_3_‐based PSCs, where SnCl_2_ served as a particularly beneficial additive. Therefore, it was proved that Cs_2_SnI_6_ could be formed with the combined action of water and oxygen. The addition of 10 mol% SnCl_2_ has hindered the oxidation on the perovskite film, thereby improving the device stability. Furthermore, they simplified the device architecture by removing the ETL without reducing device PCE (3.56%), which is beneficial to fabrication process.^[^ ^81^ ^]^ The SnI_2_ is also considered to be an effective additive to stabilize the B‐*γ*‐CsSnI_3_. Kanatzidis and co‐workers^[^ ^82^ ^]^ used excess SnI_2_ in the Sn‐based halide perovskite solar cells (CsSnI_3_) with combining a reducing atmosphere to stabilize Sn^2+^ state. During the growth of the perovskite film, excess SnI_2_ could provide the system with more Sn^2+^ and compensate for Sn^2+^ lost in the oxidization from Sn^2+^ to Sn^4+^, which would effectively reduce the p‐type conductivity. Finally, a maximum PCE of 4.81% could be achieved in the optimized CsSnI_3_ devices.

In the removal process of lead, various additives have been introduced to improve oxidation of Sn^2+^ and reduce intrinsic defects. However, both hybrid and inorganic PSCs still remain at a lower PCE level than Pb‐based PSCs. Because of low efficiency and insurmountable challenges, they have not attracted sufficient attention. Therefore, more attempts should be paid to break through the challenges.

### 2.4 State‐of‐the‐Art of Integrated Systems

#### 2.4.1 PSCs–LIBs Integrate Technology

As is known, rechargeable LIBs are the commercialized energy storage devices in the past decades. Owing to the advantages of high energy density and stable positive/negative electrode materials, LIBs could be introduced as the competitive devices in the integrated energy conversion–storage systems. Initially, LIBs were employed to integrate with Si‐based photovoltaic devices.^[^ ^83^ ^]^ With the development of photovoltaic technology, they were also used to integrate with dye‐sensitized solar cells.^[^ ^84^ ^]^ However, the output voltages of the integrated Si‐based photovoltaic technology and integrated DSSCs presented <0.7 and <0.8 V, respectively. The suppressed voltages might be linked with the insufficient capabilities to create adequate potential in a power storage system. As a result, more series units are needed to be connected, which is contradictory with the technical development of lightweight and compact integrated systems. In this aspect, further studies have demonstrated that the PSCs possess the ability to provide an output voltage above 1.0 V, and thus the integrated systems upon PSCs–LIBs could be potentially concerned. Generally, the integrated strategy between light harvesting devices and energy storage devices could be divided into three prototypes, i.e., wire connection, three‐electrode integration (shared positive or negative electrodes), and two‐electrode connection (Figure 1). In the review by Lennon and co‐workers, certain systems integrated with sensors, wearable electronics, and autonomous medical monitoring have been discussed, and the corresponding integrating strategies have been also summarized.^[^ ^14^ ^]^

**Figure 4:**
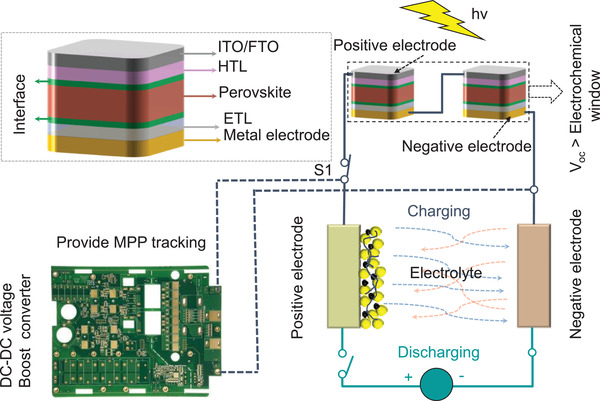
Conventional discrete charging: LIBs/supercapacitors. The traditional integration strategy is to convert and store energy by connecting PSCs and energy storage units (Li‐ion battery or supercapacity) in series through wires. Generally, the external DC–DC voltage boost converter between PSC modules and energy storage units is introduced to provide MPP tracking.

**Figure 5:**
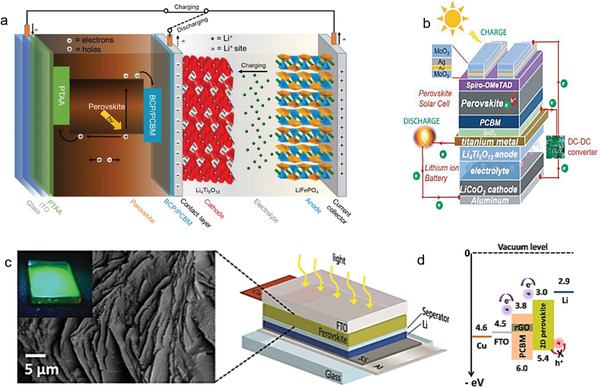
Three‐electrode and two‐electrode configurations of PSCs–LIBs. a) Schematic diagram of the fabricated system of PSCs–LIB by three‐electrode connection. Reproduced with permission.^[^ ^118^ ^]^ Copyright 2020, American Chemical Society. b) Device operation schematic. Reproduced with permission.^[^ ^88^ ^]^ Copyright 2020, Wiley‐VCH. c) SEM image of drop‐cast 2D perovskite electrodes taken at 45° tilt. The inset shows a PL image of the corresponding perovskite film (*λ*_ex_ ≈ 300 nm LED source). Schematic of perovskite photobatteries. d) Energy level diagram of perovskite photobatteries. Reproduced with permission.^[^ ^89^ ^]^ Copyright 2018, American Chemical Society.

According to the definition, wire connection is a direct and the easiest way to integrate two devices through additional conductive wires in series to achieve energy conversion and storage (**Figure** **4**). In 2015, Xu et al.^[^ ^19^ ^]^ employed four MAPbI_3_‐based PSCs (connection in series) with PCE = 12.65% (15.67% for each single cell), *V*_oc_ = 3.84 V (0.96 V for each single cell), *J*_sc_ = 4.82 mA cm^−2^ (22.85 mA cm^−2^ for each single cell), and *FF* = 0.68 (0.71 for each single cell) to directly photocharging the LIBs (LiFePO_4_ as the positive electrode and Li_4_Ti_5_O_12_ as the negative electrode). The system could deliver an overall photoelectric conversion–storage efficiency of 7.80%, with a stable self‐charge cycle under a constant illumination (with AM1.5G for 17.8 h). The results highlighted a significant progress in the development of PSC‐based hybrid integration systems. Similar integration system was also designed by Weng et al.,^[^ ^86^ ^]^ which demonstrated the high feasibility of the aforementioned wire connection strategy. Noticeably, aqueous electrolytes are easier to leak in the three‐electrode or two‐electrode integrated system. However, the aforementioned hybrid device exhibited an obvious power deterioration during constant photocharging process, and meanwhile it may lead to the decline in the life and stability of the entire system. For improving the operation, Qiao and co‐workers reported a feasible photocharging approach to charge LIBs (Li_4_Ti_5_O_12_ as the negative electrode and LiCoO_2_ as the positive electrode) with a single MAPbI_3_‐based PSCs (PEC = 14.4%; *V*_oc_ = 0.96 V; *J*_sc_ = 21.71 mA cm^−2^; *FF* = 0.68), showing an ultralow power direct current–direct current (DC–DC) boost converter.^[^ ^86^ ^]^ This DC–DC converter can provide maximum power point (MPP) tracking for the PSCs devices along with overcharge protection for the LIBs. An overall efficiency of 9.36% and average storage efficiency of 77.2% was achieved in this approach. Although the wire‐connection stacking prototype demonstrated effectiveness, it seems incompatible to meet the requirements of flexibility, lightweight, and compactness in the mobile devices. More importantly, there would be a portion of current loss from the connecting cables. Therefore, the key factors including flexibility, compactness, lightness, mobility, easy installation, and wide applications are highly necessary, which can be achieved by a shared electrode strategy (either three‐electrode or two‐electrode). In addition to these advantages, area match between PSCs and LIBs is also important to optimize the maximum power output. For example, Kin et al.^[^ ^87^ ^]^ designed a three‐electrode integrated system (with a shared positive electrode) via DC–DC boost converter. Combined with a single PSCs and battery, the as‐integrated system provided an overall efficiency of 9.8% (**Figure** **5**a). They demonstrated that the boost converter resulted in a constant voltage over time. Meanwhile, the system could convert an almost constant power input to the battery cell in different area‐matched integrated systems (0.64 and 0.9 cm^2^). Gurung et al.^[^ ^88^ ^]^ reported a similar three‐electrode integrated configuration (with a shared negative electrode), which consisted of a LIB (positive electrode: LiCoO_2_; negative electrode: Li_4_Ti_5_O_12_) along with PSCs on the top (PCE = 10.96%; *V*_oc_ = 1.09 V; *J*_sc_ = 15.45 mA cm^−2^; *FF* = 0.656). Particularly, a common Ti metal substrate was employed between the LIBs and PSCs (Figure 5b). Meanwhile, the DC–DC boost converter provided efficient manipulation on the battery management and maximum power point tracking. In this integrated system, an overall photoelectric conversion–storage efficiency of 7.3% along with light charging cycle performance (30 cycles) was achieved. In addition, two‐electrode integrated system (2D (C_6_H_9_C_2_H_4_NH_3_)_2_PbI_4_)/reduced graphene oxide (rGO)/poly(vinylidene fluoride) (PVDF) as the positive electrode and Li metal as the negative electrode) was also successfully fabricated by Ahmad et al.^[^ ^89^ ^]^ In this integrated system, perovskite thin film (2D (C_6_H_9_C_2_H_4_NH_3_)_2_PbI_4_) could act as the role for both energy generation and storage (Figure 5c,d), and the corresponding possible mechanism for energy conversion and storage was described as following

(6) $$\begin{array}{ccc} 2\mathrm{Li} & + & {\left(C_{6}H_{9}C_{2}H_{4}NH_{3}\right)}_{2}\mathrm{Pb}I_{4}\;\to \;2\left(C_{6}H_{9}C_{2}H_{4}NH_{3}I\right)\\ & + & 2\mathrm{LiI}\;+\;\mathrm{Pb}\left(m\right) \end{array}$$

This appears an ideal compacted solution for energy generation and storage. However, there is a strict requirement on the compatibility of active materials in such system. Additionally, the light absorptivity and stability of the perovskite film should be also concerned for long‐term cycle of energy conversion and storage. Recently, some semiconductor materials (VO_2_, V_2_O_5_, g‐C_3_N_4_, organic molecules (tetrakislawsone (TKL)), etc.) with suitable bandgaps have been employed as both electrode active materials and photosensitizers in the two‐electrode systems.^[^ ^90^ ^]^ Light‐excited electrons can be output to the external circuit through the electron transmission medium during the charging process. Thus, the rate performance and energy density of the energy storage units could be significantly enhanced via the effect of photoelectrochemistry.

According to the integrated system upon PSCs–LIBs, there are sufficient studies to illustrate the feasibility and challenges of the integrated system. First, the wire connection could increase the package and energy loss of the integrated system, and it is difficult to achieve the maximum power matching between PSCs and LIBs. Although introduction of DC–DC boost converter ensures the direct power output of single PSCs, the original design could be varied. In fact, it seems the most stable device up to date. In addition, rational design of the shared electrode is the key in the three‐electrode integration, which is related to the efficient transportation of electrons. Finally, both the thermal and ambient stability should be another key technical requirement for the long‐term stable integrated system upon PSCs–LIBs, because such parameter is substantially responsible for stable power output and efficient energy storage and utilization.

#### 2.4.2 Integrated Technology for PSCs–Supercapacitors

Apart from the important performance factors, the poor energy and power density of the integrated systems are also crucial for the overall efficiency of the device. It is well known that LIBs possess a high energy density, while supercapacitors presented high power density. Furthermore, the ultralong cycling stability of supercapacitors (commercial products more than 100 000 cycles) is also greater than the other energy storage devices. More importantly, supercapacitors in the integrated systems are commonly assembled with carbon‐based electrodes (carbon nanotubes, graphene, carbon composites, etc.), which can be also used as the back and front contact layers in the PSCs. Because of the hydrophobic nature and chemical stability of the carbon derivatives, the mechanical and chemical endurance of the device can be ensured (Figure 4). Actually, various types of off‐grid electrochemical capacitors have been reported, such as piezosupercapacitors, optically rechargeable supercapacitors, thermally rechargeable supercapacitors, and integrated triboelectric nanogenerators and supercapacitor systems.^[^ ^91^ ^]^ In this section, we would mainly focus on the PSCs–supercapacitors.

Li et al.^[^ ^92^ ^]^ fabricated a flexible self‐powered system for strain sensors, which was composed of a flexible PSC module (four flexible PSCs connected in series by silver wires), flexible lithium‐ion hybrid capacitor (LIHC) module, and a graphene‐based strain sensor. As an energy conversion, tandem PSCs can easily deliver a remarkable voltage output of 3.95 V and a high PCE of 10.20%, which is sufficient to charge the integrated LIHCs. Accordingly, the LIHCs device (Li_4_Ti_5_O_12_/reduced graphene oxide as the negative electrode and activated carbon as the positive electrode) delivered a favorable energy/power density (60.2 Wh kg^−1^/50 W kg^−1^ and 40 Wh kg^−1^/2000 W kg^−1^). Meanwhile, the flexible integrated system of PSCs–LIHCs could present an overall efficiency of 8.41% at a discharge current density of 0.1 A g^−1^. Using the same integration approach, Xu et al.^[^ ^93^ ^]^ reported an integrated system with a CH_3_NH_3_PbI_3_‐based PSCs connected by a bacterial supercapacitor, which was assembled with cellulose membrane/polypyrrole (PPy) nanofibers/multiwalled carbon nanotubes (MWCNTs). The hybrid devices exhibited a high energy storage efficiency (10%) and output voltage of 1.45 V, with low interruptions in the cycles. However, active area mismatch between the supercapacitors and solar cells would result in a long charging time (300 s). Different from the above cases, Du et al.^[^ ^94^ ^]^ reported a flexible all‐solid‐state wire‐connection integrated device upon PSCs/supercapacitors. In this device, CH_3_NH_3_PbI_3−_ *_x_* Cl*_x_* was employed as the light absorbing layer in the PSCs, and the self‐stacked solvated graphene (SSG) film simultaneously plays as the positive electrode and negative electrode in the supercapacitors. The strategy of using solid electrolytes greatly reduced the degradation that are induced from the leakage of aqueous electrolyte, and such strategy may meet the technical requirements of integrated systems.

**Figure 6:**
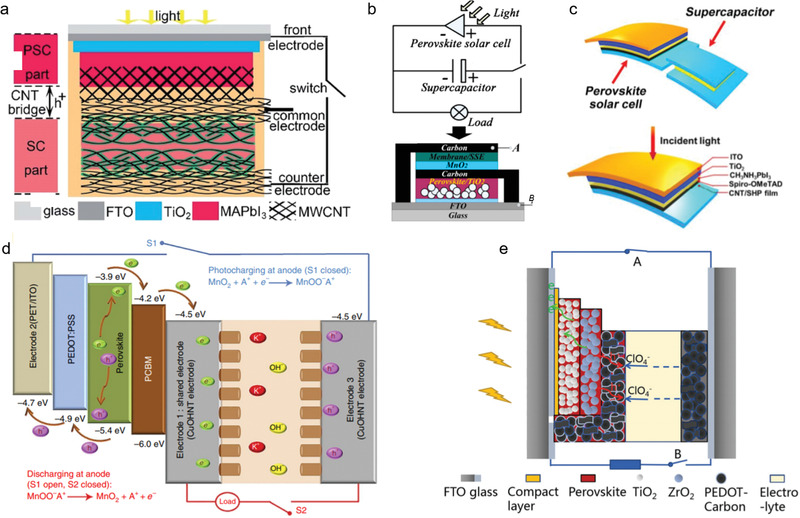
Three‐electrode configuration: supercapacitors. a) Schematic of the photocapacitor and energy level schematic. Reproduced with permission.^[^ ^95^ ^]^ Copyright 2017, The Royal Society of Chemistry. b) Schematic diagram and structural schematic of the integrated device connected in parallel. Cross‐sectional SEM image of the integrated device; inset: the close‐up of the PSCs part. Reproduced with permission.^[^ ^96^ ^]^ Copyright 2017, American Chemical Society. c) Schematic illustration and photocharging. Schematic illustration of a fusible perovskite solar cell. Reproduced with permission.^[^ ^97^ ^]^ Copyright 2015, The Royal Society of Chemistry. d) Charge transfer mechanism of the combination device. Reproduced with permission.^[^ ^98^ ^]^ Copyright 2016, Nature Publishing Group. e) Schematic illustration and work mechanism of the photosupercapacitor device constructed based on a printable perovskite solar cell. Reproduced with permission.^[^ ^99^ ^]^ Copyright 2016, Wiley‐VCH.

In addition to the direct stacking integration solution, the design strategy on the shared electrode is still an important development direction. Liu et al.^[^ ^95^ ^]^ integrated an all‐solid‐state photocharging capacitor based on PSCs (CH_3_NH_3_PbI_3_) and supercapacitors (polyaniline (PANI)/carbon nano tube (CNT)), in which a CNT bridge was employed to inhibit the water from the aqueous gel electrolytes (**Figure** **6**a). Under fluctuating sunlight, the hybrid device exhibited a specific area capacitance of 422 mF cm^−2^ with a Coulombic efficiency ≈96% and energy storage efficiency ≈70.9%. However, the overall efficiency of 0.77% is even lower than that of some wire‐connected devices, which may be attributed to the low energy conversion efficiency and deterioration of PSCs. Liu et al.^[^ ^96^ ^]^ designed a similar hybrid device (PSCs–supercapacitors) via combining photoelectric conversion and energy storage with a shared carbon electrode. Such shared electrode served as both the cathode for PSCs (PCE = 7.79%) and the anode for MnO_2_‐based supercapacitors (Figure 6b). When the supercapacitor was charged by the PSCs under the AM1.5G white light illumination (0.071 cm^2^ active area, a 0.84 V voltage, and a 76% energy storage), an overall conversion efficiency of ≈5.26% could be achieved. In the study by Sun et al.,^[^ ^97^ ^]^ an integration structure upon PSCs–supercapacitors was fabricated by incorporating the electrically conducted carbon nanotube and a self‐healing polymer (CNT/SHP) film electrode (Figure 6c). In addition to the shared carbon electrode, electrically conductive metal electrode was also regarded as a promising shared integrated electrode, according to the report by Li et al.^[^ ^98^ ^]^ They proposed an integrated system with connecting a PSCs on the top of a symmetric supercapacitor via an all‐solid‐state copper ribbon for energy harvesting and storage (Figure 6d). This Cu ribbon can serve as the shared electrode for the system and also as an electrode for generating copper hydroxide nanotubes (CuOHNT) in the supercapacitor. Hybrid ribbons upon PSCs–supercapacitors can be woven into a textile to form supporting cotton yarns. When the solar ribbon is illuminated by the simulated solar light, the supercapacitor shows an energy density of 1.15 mWh cm^−3^ and a power density of 243 mW cm^−3^. Xu et al.^[^ ^99^ ^]^ also reported a three‐electrode integrated hybrid device using a shared electrode of poly(3,4‐ethylenedioxythiophene) (PEDOT)–carbon. In this electrode, PEDOT–carbon was employed as the positive electrode in the PSCs and symmetric supercapacitors (Figure 6e). In this system, the overall efficiency and energy storage efficiency was 4.70% and 73.77%, respectively. However, the efficiency can be influenced by both the PSCs and working function of PEDOT–carbon electrode. Zhou et al.^[^ ^100^ ^]^ reported a perovskite (CH_3_NH_3_PbI_3−_ *_x_* Cl*_x_*) photovoltachromic supercapacitor with all‐transparent electrodes by coanode (MoO_3_) and/or cocathode (WO_3_). Such hybrid system provides an integration of energy harvesting and storage device, an automatic and wide‐color smart switch, and enhanced photostability of PSCs. Along with energy storage process, the color could change from semitransparent to dark‐blue. Because the colored PSCs–supercapacitors blocks off most of the illuminated light, the photocharging process could be automatically switched off. The PCEs of the coanode and cocathode PSCs–supercapacitors dropped to 3.73% and 2.26%, respectively.

Consequently, supercapacitors are energy storage devices with high power density. In most cases, carbon materials are used as the working electrode for symmetrical or asymmetrical supercapacitors due to the obvious advantages, such as high‐power density, great chemical stability, high flexibility, low mass, and high conductivity. The shared electrode is an important factor for the integration system upon PSCs–supercapacitors. More importantly, the carbon exhibits a hydrophobic characteristic due to excellent negative surface zeta index. This would guarantee the stability of perovskite layers in the presence of oxygen and water.^[^ ^101^ ^]^ In contrast, metals or metal oxides were employed as the coelectrode of the PSCs–supercapacitors, which would suffer severe degradation from interfacial chemical conversion. Therefore, carbon materials are promising candidates, while they would possess a low energy density.

#### 2.4.3 Integration Technology upon PSCs–Other Energy Storage Devices

Many efforts have been employed to the development of high‐performance integrated energy conversion–storage systems to meet the diverse energy demands, while both high power density and high energy density are still required. However, the state‐of‐the‐art of the integrated energy conversion–storage systems upon LIBs suggests that there are fundamental limitations under high charge–discharge rate, due to the limited rate performance of LIBs. To address these issues, considerable efforts have been devoted to design combined systems upon PSCs and supercapacitors. Currently, improved power density could be obtained, while low operation voltage (<0.8 V) and limited energy density (usually as low as 15 Wh kg^−1^) are also the shortage.^[^ ^102^ ^]^

**Figure 7:**
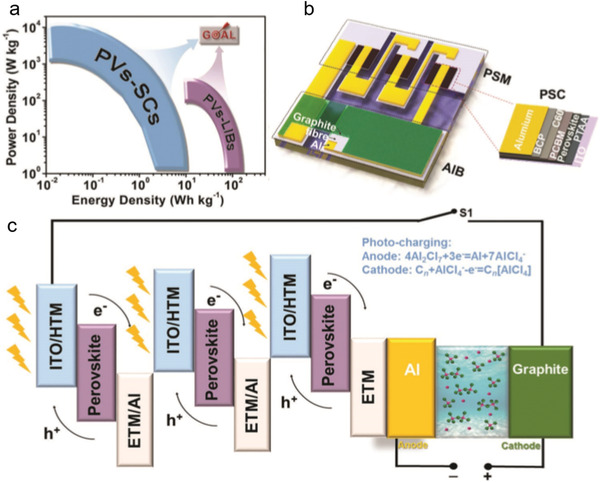
Three‐electrode configuration: AIBs. a) Schematic summary of reported integrated systems. b) Diagram of the integrated PSM–AIB solar‐rechargeable battery and a single PS. c) Device structure of the integration system with the photocharging mechanism. Reproduced with permission.^[^ ^104^ ^]^ Copyright 2019, Wiley‐VCH.

Alternatively, emerging aluminum‐ion batteries (AIBs) with fast charge–discharge feature demonstrate a new direction to realize both high power density and high energy density.^[^ ^103^ ^]^ In addition, abundant natural resources and good safety feature would allow AIBs for serving as a promising candidate beyond LIBs. In the recent attempt, Hu et al.^[^ ^104^ ^]^ designed the integrated energy conversion–storage systems by integrating tandem PSCs (MAPbI_3_; PCE = 18.5%, V_MPP_ = 2.62 V) and graphite‐based AIBs on a shared aluminum electrode without any external circuit (**Figure** **7**). With the charging voltage of AIBs, the rationally matched maximum power voltage of the tandem PSCs could reach a voltage ratio of *V*_MPP_/*V*_Battery Charging_ = 1.09, along with excellent solar‐charging efficiency ≈15.2% and a high overall efficiency ≈12.04%. The results apparently provided a novel platform for advancing portable integrated energy conversion–storage systems. Therefore, the integrated system upon PSCs–AIBs is a promising energy conversion–storage strategy. In the future, more efforts should be paid to boosting such technology in a more practical form.

**Figure 8:**
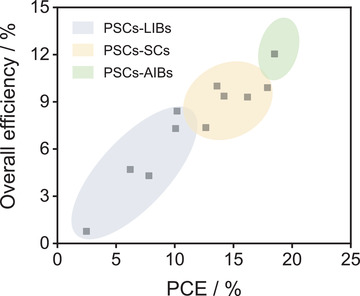
The development of energy conversion–storage integrated system in recent years.

Moreover, the PCE and overall efficiency of the integrated system upon energy conversion–storage should be substantially improved. Because the PSCs–supercapacitors are still at the developing level (**Figure** **8** and **Table** **3**), the integrated systems upon PSCs–AIBs have exhibited substantial potentials.

**Table 3:** Several of reported integrated systems based on perovskite solar cells and energy storage units

Configuration of PSCs	Features of PSC	Configuration of energy storage units^a)^	Integrated strategy	Operating voltage window	Photocharge capacity [mA g^−1^]	Photocharge energy density	*η*_overall_ [%]	Refs.
ITO/PEDOT:PSS/CH_3_NH_3_PbI_3_/PC61BM/Ca/Al	*J*_c_: 4.82 mA cm^–2^ *V*_oc_: 3.84 V *FF*: 0.68 PCE: 12.65%	PE: Li_4_Ti_5_O_12_ NE: LiFePO_4_	Wire connection	1.0–2.6 V	140.4 at 0.5C	–	7.8	^[^ ^19^ ^]^
ITO/TiO_2_/Cs_0.05_FA_0.81_MA_0.14_PbI_2.55_Br_0.45_/spiro‐OMeTAD/MoO_3_/Ag	*J*_c_: 9.44 mA cm^–2^ *V*_oc_: 2.2 V *FF*: 0.78 PCE: 16.2%	PE: LiTi_2_(PO_4_)_3_ NE: LiMn_2_O_4_	Wire connection	0.2–1.9 V	132 at 2C	–	9.3	^[^ ^85^ ^]^
ITO/PEDOT:PSS/CH_3_NH_3_PbI_3_/xstPC61BM/Ag	*J*_c_: 21.99 mA cm^–2^ *V*_oc_: 0.96 V *FF*: 0.67 PCE: 14.2%	PE: Li_4_Ti_5_O_12_ NE: LiCoO_2_	Three‐electrode	1.0–3.14 V	151.3 at 0.5C	–	9.36	^[^ ^86^ ^]^
ITO/PTAA/MAPbI_3_/PCBM/BCP/Ag	*J*_c_: 19.8 mA cm^–2^ *V*_oc_: 1.24 V *FF*: 0.731 PCE: 17.9%	PE: Li_4_Ti_5_O_12_ NE: LiFePO_4_	Three‐electrode	0.5–2.9 V	0.372 mAh at 6C	–	9.9	^[^ ^87^ ^]^
FTO/SnO_2_/PCBM/MAPbI_3_/spiro‐OMeTAD/Ag	*J*_c_: 15.45 mA cm^–2^ *V*_oc_: 1.09 V *FF*: 0.658 PCE: 10.96%	PE: Li_4_Ti_5_O1_2_ NE: LiCoO_2_	Three‐electrode	1.0–3.14 V	155.2 at 1C		6.78	^[^ ^150^ ^]^
FTO/rGO (or PCBM)/C_6_H_9_C_2_H_4_NH_3_)_2_PbBr_4_/Cu	–	PE: C_6_H_9_C_2_H_4_NH_3_)_2_PbBr_4_ NE: Li	Two‐electrode	1.4–3.0 V	100		0.034	^[^ ^89^ ^]^
ITO/NiOx/MA_1−_ *_y_* FA*_y_* PbI_3−_ *_x_* Cl*_x_*/PCBM/BCP/Ag	*J*_c_: 18.71 mA cm^–2^ *V*_oc_: 1.05 V *FF*: 0.71 PCE: 14.01%	PE: Li_4_Ti_5_O_12_/rGO NE: activated carbon	Wire connection	0–3.0 V	–	0.802 mWh at 0.1 A g^−1^	8.41	^[^ ^92^ ^]^
FTO/TiO_2_/MAPbI_3_/spiro‐OMeTAD/Au	*J*_sc_: 19.90 mA cm^−2^ *V*_oc_: 0.974 V *FF*: 0.7 PCE: 13.6%	BC/PPy nanofibers/MWCNT films	Wire connection	0–0.71 V	572 mF cm^−2^ at 1 mA cm^−2^	–	10	^[^ ^93^ ^]^
ITO/PEDOT:PSS/MAPbI_3−_ *_x_* Cl*_x_*/PC61BM/Al	*J*_sc_: 22.59 mA cm^−2^ *V*_oc_: 0.9 V *FF*: 0.695 PCE: 14.13%	Self‐stacked solvated graphene (SSG)	Wire connection	0–0.9 V	45 s discharge time at 1.0 A g^−1^	–	–	^[^ ^94^ ^]^
FTO/TiO_2_/MAPbI_3_/MWCNT	*J*_sc_: 9.645 mA cm^−2^ *V*_oc_: 0.711 V *FF*: 0.396 PCE: 2.13%	PANI/CNT	Three‐electrode	0–0.7 V	422 mF cm^−2^ at 103.4 F g^−1^	–	0.76–0.77	^[^ ^95^ ^]^
FTO/TiO_2_/MAPbI_3_/carbon	*J*_sc_: 15.7 mA cm^−2^ *V*_oc_: 0.96 V *FF*: 0.52 PCE: 7.79%	PE: MnO_2_ NE: mesoporous carbon film	Three‐electrode	0–1.0 V	–	–	5.26	^[^ ^96^ ^]^
ITO/PEDOT:PSS/MAPbI_3_/PCBM/CuOHNT	*J*_sc_: 16.44 mA cm^−2^ *V*_oc_: 0.96 V *FF*: 0.66 PCE: 10.41%	CuOHNT	Three‐electrode	0–0.8 V	227.58 F g^−1^ at 1 mA cm^−2^	1.82 mWh cm^−3^ at 1 mA cm^−2^		^[^ ^98^ ^]^
FTO/TiO_2_/MAPbI_3_/PEDOT–carbon	*J*_sc_: 18.62 mA cm^−2^ *V*_oc_: 0.71 V *FF*: 0.48 PCE: 6.37%	PEDOT–carbon	Three‐electrode	0–0.7 V	11.5 mF cm^−2^ at 1 mA cm^−2^	–	4.7	^[^ ^99^ ^]^
FTO/TiO_2_/MAPbI_3−_ *_x_* Cl*_x_*/spiro‐OMeTAD/MAM	*J*_sc_: 18.17 mA cm^−2^ *V*_oc_: 1.0 V *FF*: 0.654 PCE: 11.89%	WO_3_	Three‐electrode	0–1.0 V	459.6 F m^−2^	35.9 mWh m^−2^ at 0.1 mA cm^−2^	7.8	^[^ ^100^ ^]^
ITO/PTAA/MAPbI_3_/PCBM/Al	*J*_sc_: 7.65 mA cm^−2^ *V*_oc_: 3.28 V *FF*: 0.735 PCE: 18.5%	PE: graphite NE: Al	Three‐electrode	0.4–2.4 V	78 mAh g^−1^ at 41 mA g^−1^	43 Wh kg^−1^	12.56	^[^ ^104^ ^]^

^a)^ PE: positive electrode; NE: negative electrode.

In addition to AIBs, smart electrochromic window is also a promising technology, which provides multifunction support in solar energy harvesting, storage and reutilization of PSCs. For instance, Tu and co‐workers^[^ ^105^ ^]^ reported a wire‐connected integrated system based on perovskite solar cell (FTO/TiO_2_/ZrO_2_/MAPbI_3_/carbon) and it could be used for powering solid‐state electrochromic batteries, with application in smart windows. In the energy storage unit, an rGO‐connected bilayer NiO nanoflake array and a WO_3_ nanowire array were employed in the positive electrode and negative electrode, respectively. The electrochromic battery presented fast optical switching ability, within 2.5 s for coloring (charge process) and 2.6 s for bleaching (discharge process). Smart windows based on photovoltaics offer great interesting in semitransparent windows, colorful wall facades, electrochromic windows, and thermochromic windows.^[^ ^106^ ^]^ Note that heat would be generated during the photon–electron conversion process due to the thermodynamic relaxation in the short wavelength range, which is harmful to PSCs and integrated systems.^[^ ^107^ ^]^ To address this problem, Lin and co‐workers have recently proposed an integrated system by connecting tandem PSCs with a thermoelectric device. In such system, additional thermoelectric energy induced by temperature difference between the solar cell and environment could be stored.^[^ ^108^ ^]^ Apparently, other types of photovoltaic technologies could be also integrated with thermoelectric devices.^[^ ^109^ ^]^

## 3 Overall Critical Challenges

Besides the cost of commercialized products, the parameters of integration strategy, stability, and energy density are the three critical concerns in the integrated energy conversion–storage systems.

### 3.1 Integration Strategies

There are three integration strategies that are reviewed in Section 2.4, such as independent connection (i.e., wire connection), three‐electrode and two‐electrode configurations. All the structures are designed for achieving flexibility, compactness, lightness, and easy assembly. The functional applications, geometry, and size of the entire hybrid devices are determined by each unit.

Generally, LIBs, supercapacitors, AIBs, and other forms of electrochemical energy storage units are assembled based on the liquid electrolytes (with a feature of high ionic conductivity). The hydration or decomposition of the liquid electrolytes would undermine the stability of the perovskite films. From this perspective, the wire connection seems to be more suitable for liquid energy storage units, while this would constrain the applications of PSCs. On the other hand, wire connection is a traditional integration strategy, which is contrary to compactness and lightness in the integrated energy conversion–storage systems. A feasible development direction is the integration of all solid‐state units.

A three‐electrode combination is commonly introduced in the construction of photocapacitors and photobatteries. In this complicated system, a functional layer (as the shared positive electrode or shared negative electrode) is a critical factor, which would inject photoexcited carriers from the PSCs into the electrochemical energy storage system. This requires the shared electrode to possess excellent conductivity and stability. Theoretically, carbon‐based electrodes or appropriate alkali metals are feasible as the shared electrode for LIBs, supercapacitors, and AIBs. Therefore, chemically stable and safe carbon electrodes seem more suitable candidates, while the low energy density of graphite electrodes is difficult to meet actual demands.

Alternatively, the high thermal conductivity of the shared electrode is necessary to evacuate the heat generated by light radiation and avoid the degradation of the perovskite light‐absorbing layer. Simultaneously, this behavior will vary the working temperature of the electrochemical process. Therefore, the thermal dissipation function of the entire integrated system should be considered.

In addition to the three‐electrode system, the two‐electrode integration is the most attractive integration strategy. The perovskite light‐absorbing layer can simultaneously convert and harvest energy. Although the space utilization can be maximized, it is difficult to achieve a balance between the electrochemical process and light conversion process.

### 3.2 Overall Efficiency

Overall efficiency is an important criterion for evaluating the high‐performance integrated energy conversion–storage systems. However, the highest overall efficiency with lab‐scale PSCs–AIBs delivered 12.04% for a three‐electrode system, which is not sufficient to meet commercialization of integrated energy conversion–storage systems. The overall efficiency of integrated energy conversion–storage systems refers to the conversion efficiency of PSCs and storage efficiency of the batteries. The storage efficiency was determined by the electrode and electrolyte, and therefore it is important to choose a reliable electrochemical system in the integrated devices.

Note that the integrated energy conversion–storage systems are needed to operate at the maximum power of the PSCs, in order to achieve the maximum overall efficiency. Namely, the *V*_oc_ of PSCs must be higher than the maximum electrochemical window of the energy storage, which enables for full charging. Therefore, the power matching between PSCs and electrochemical energy storage units is a key factor. Generally, the voltage ratio of the maximum *V*_oc_ (PSCs) to the maximum charging voltage approaches 1.0, which suggests an efficient maximum power tracking. On the contrary, it would result in overcharging or undercharging in the battery and increase the rate of the battery aging and stability degeneration. In addition, the maximum power tracking (MPPT) by DC–DC converter is a feasible strategy to improve power matching. The PSCs are connected to power electronic units with charge controllers and inverters, which are combined with the maximum power tracking. By this way, flexible selection of the integrated units could be available. For example, the current of low‐*V*_oc_ PSCs can be used to charge high‐voltage electrochemical batteries through converter, which indicates that insufficient voltage will be compensated by MPPT. This greatly improves the adaptability, safety, and stability of the energy storage units for stabilizing the power output. However, the use of DC–DC converters limits the integrated structure of PSCs and energy storage units, which implies that independent connection is different in a complicated integration.

### 3.3 Overall Stability

The stability is a significant factor for long‐term operation in the integrated energy conversion–storage systems, which involves the photostability of the perovskite films, electrochemical stability of the energy storage unit, and thermal stability of the solar units. Note that the integrated energy conversion–storage systems are highly dependent on the stability of PSCs, and therefore the stability of PSCs is a prerequisite for the long‐term stable operation.

For the photostability in the perovskite films, the environmental factors, such as H_2_O, temperature, O_2_, etc., are the main challenges. In recent years, numerous improvement strategies have been made to enhance the stability of perovskite films (both hybrid PSCs and all‐inorganic PSCs). Typical strategies include interface engineering,^[^ ^110^ ^]^ additive engineering,^[^ ^111^ ^]^ 2D/3D perovskite design strategy,^[^ ^112^ ^]^ metal cation doping strategy,^[^ ^113^ ^]^ and defect passivation strategy.^[^ ^114^ ^]^ Similarly, inevitable phase transition (from *α* phase to *δ* phase) is a critical obstacle in the all‐organic perovskite solar cells. Two potential strategies, i.e., doping engineering and quantization, are promising to overcome the challenges of phase transition and water intrusion.^[^ ^115^ ^]^ Fortunately, these progresses can be directly incorporated into the integrated system, and PSCs are expected to be commercialized with further efforts.

Subsequently, the integrated energy conversion–storage systems should possess favorable power matching, and the energy harvesting units are required to present stable power output. Simultaneously, higher requirements should focus on improving the battery performance. To date, the low‐power output in a single PSC is difficult to support electrochemical battery of high working potential. However, a general integration strategy is utilization of series connection with single PSC to provide required charging power, which inevitably increases the size and integration complexity of the target systems.

Another challenge in the integrated energy conversion–storage systems is the thermal generation induced by light radiation. The studies on the solid electrolytes in the electrochemical batteries received much attention, aiming to solving safety and promoting energy density. Therefore, employment of the all‐solid‐state batteries can not only avoid the influence of volatile and corrosive liquid electrolytes on the stability of PSCs, but also is beneficial to significantly promote the thermal stability and safety of the integrated systems.

### 3.4 Energy and Power Density

To meet application demands, pursuit of high energy and power density is an essential challenge in the integrated energy conversion–storage systems. Actually, the traditional LIBs assembled with Li metal (500 Wh kg^−1^) or Si‐based (400 Wh kg^−1^) negative electrodes possess high theoretical energy density.^[^ ^116^ ^]^ For achieving high energy density of the electrochemical batteries, LIBs are promising energy storage units in the integrated systems. However, the deposition/stripping processes of Li^+^ on the negative electrode would lead to inevitable lithium dendrites or volume expansion in the Si crystals. Thus, such unexpected behaviors would cause severe crisis in battery safety and capacity decay. On the other hand, power density is another crucial technical requirement in the integrated systems. As typical examples, supercapacitors and AIBs are regarded as the devices with high power density.^[^ ^103^, ^117^ ^]^ Particularly, AIBs exhibited safe advantage in the grid‐scale energy storage. Therefore, integration of PSCs with supercapacitors or AIBs is an effective approach to meet the applications where power density is a requirement.

On the other hand, the roll‐to‐roll battery structure is difficult to match with the planar structure of the PSCs. Normally, energy storage units are needed to be integrated into the surface of the PSCs, and thus a highly integrated structure could be achieved. Apparently, the current effective area of PSCs is not sufficient to support a compact integrated structure design. Therefore, the large‐area fabrication of perovskites is a significant technique for improving the overall energy density.

## 4 Conclusions and Perspectives

In summary, increasing development of solar energy is expected to deliver a fossil fuel‐free energy market in the foreseeable future. In this perspective, PSCs have received considerable attention due to their great progresses in the PCE, which can compete with Si‐based solar cells. According to the current PSCs, it is expected that there will be substantial breakthroughs in the future. However, solar cells are the intermittent devices that enable to convert sunlight into electricity without harvesting energy. In the context of the current energy crisis, therefore, the integration of solar cells and energy storage devices is an important strategy. As a clean and renewable energy source, however, it is difficult to achieve improved PSCs due to severe challenges, such as unstable power output and high safety risk. Thus, all‐inorganic perovskite is expected to increase the thermal stability of the hybrid solar cells. Up to date, no meaningful progress has been made in promoting the efficiency, although stability has been greatly improved.

In order to in‐depth understanding of perovskite solar cells, in this review, we have also discussed the operation mechanism, key parameters (affecting PCE), critical problems, and challenges of PSCs in details. This would trigger the development and applications of energy conversion and storage. The integrated energy conversion–storage systems could be considered as the derivative technology of PSCs, which rely on the technical advantages of PSCs. We have also reviewed and discussed the recent preliminary explorations in this field (in Section 2), which demonstrates the feasibility of the integrated energy conversion–storage systems. However, there are still essential challenges, including compatibility, compactness, suitable power matching, and stable power output. In the power output, it is difficult to achieve high‐potential energy storage devices due to the low output voltage of a single perovskite solar cell. Compared with simple series connection (line connection), the two‐terminal perovskite solar cells or PSCs/Si configurations greatly increases the output voltage, while the overall occupied volume could be reduced. In addition, the two‐electrode integrated design possesses the most advantages among the feasible integrated systems, in which the perovskite thin film plays a critical role in generating and storing the electrical energy. Under solar radiation (100 mW cm^−2^), the coupling process of photoelectron excitation and electrochemistry enhances the storage efficiency and power density of the integrated system. Thereby, high‐efficiency integration of light energy harvesting and storage could be realized.

**Figure 9:**
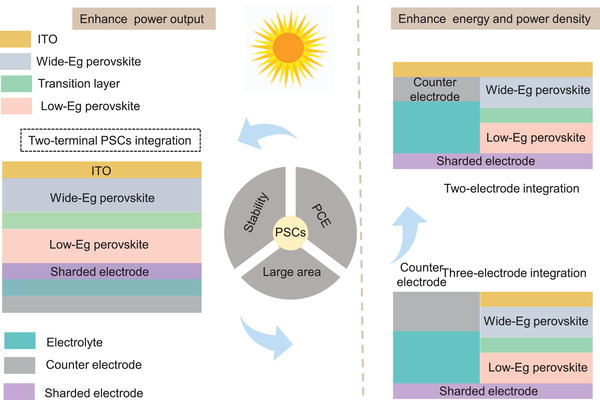
Future development direction and prospects of perovskite solar cells and integrated energy conversion–storage systems.

In the attempt of improving the overall efficiency of the integrated energy conversion–storage systems, great contribution has been made up to date, because overall efficiency is one of the most significant factors. However, limited attention has been paid to the other parameters, i.e., energy density and power density of the overall charge storage. As an integrated system, it is difficult to meet the demands in energy density and power density if the optimization is solely applied to the active materials or electrolytes. The photorechargeable battery is an energy storage device, in which both generation of light‐excited charge carriers and electrochemical reaction proceed simultaneously. The additional photoelectrons will further enhance the energy and power density of the batteries. Meanwhile, we suggest an integration system for photochargeable batteries and PSCs, which is expected to achieve the goal of maximizing the overall energy and power density. In this system, the design of high transmission positive electrodes (alkali or nonalkali metal electrodes as negative electrodes) is the key criterion. According to the fundamental of PSCs, the active materials of the positive electrodes should be the appropriate semiconductors with good chemical stability (in the electrolytes), strong light absorption (matched bandgap), and high energy density. Whereby, the PSCs and energy storage units can harvest light simultaneously, and the integrated energy conversion–storage systems is self‐charged. More importantly, the overall energy density and power density could be substantially enhanced (**Figure** **9**).

## Conflict of Interest

The authors declare no conflict of interest.

## References

[1] A. Hagfeldt , G. Boschloo , L. Sun , L. Kloo , H. Pettersson , Chem. Rev. 2010, 110, 6595. 20831177 10.1021/cr900356p

[2] K. A. Mazzio , C. K. Luscombe , Chem. Soc. Rev. 2014, 44, 78. 25198769 10.1039/c4cs00227j

[3] a) Q. Li , Y. Liu , S. Guo , H. Zhou , Nano Today 2017, 16, 46;

[4] a) J. Zhao , A. Wang , M. A. Green , F. Ferrazza , Appl. Phys. Lett. 1998, 73, 1991;

[5] K. A. Munzer , K. T. Holdermann , R. E. Schlosser , S. Sterk , IEEE Trans. Electron Devices 1999, 46, 2055.

[6] a) M. A. Green , J. Mater. Sci.: Mater. Electron. 2007, 18, 15;

[7] a) H. Águas , S. K. Ram , A. Araújo , D. Gaspar , A. Vicente , S. A. Filonovich , E. Fortunato , R. Martins , I. Ferreira , Energy Environ. Sci. 2011, 4, 4620;

[8] a) A. Richter , M. Hermle , S. W. Glunz , IEEE J. Photovoltaics 2013, 3, 1184;

[9] A. Mavlonov , T. Razykov , F. Raziq , J. Gan , J. Chantana , Y. Kawano , T. Nishimura , H. Wei , A. Zakutayev , T. Minemoto , Sol. Energy 2020, 201, 227.

[10] X. Zhao , R. Ma , M. Yang , H. Yang , P. Jin , Z. Li , Y. Fan , A. Du , X. Cao , J. Alloys Compd. 2017, 726, 593.

[11] a) L. Hong , Rusli , X. Wang , H. Zheng , L. He , X. Xu , H. Wang , H. Yu , J. Appl. Phys. 2012, 112, 054326;

[12] a) J. Meakin , R. Birkmire , L. Dinetta , P. Lasswell , J. Phillips , Sol. Cells 1986, 16, 447;

[13] a) Q. Liu , J. Wang , Sol. Energy 2019, 184, 454;

[14] D. Lau , N. Song , C. Hall , Y. Jiang , S. Lim , I. Perez‐Wurfl , Z. Ouyang , A. Lennon , Mater. Today Energy 2019, 13, 22.

[15] a) P. Caprioglio , M. Stolterfoht , C. M. Wolff , T. Unold , B. Rech , S. Albrecht , D. Neher , Adv. Energy Mater. 2019, 9, 1901631; 10.1002/adma.201902762 31631441

[16] Z. Li , T. R. Klein , D. H. Kim , M. Yang , J. J. Berry , M. F. van Hest , K. Zhu , Nat. Rev. Mater. 2018, 3, 18017.

[17] a) Q. Dong , Y. Fang , Y. Shao , P. Mulligan , J. Qiu , L. Cao , J. Huang , Science 2015, 347, 967; 25636799 10.1126/science.aaa5760

[18] a) W. Guo , X. Xue , S. Wang , C. Lin , Z. L. Wang , Nano Lett. 2012, 12, 2520; 22519631 10.1021/nl3007159

[19] J. Xu , Y. Chen , L. Dai , Nat. Commun. 2015, 6, 810. 10.1038/ncomms9103 PMC4560813 26311589

[20] a) M. M. Byranvand , T. Kim , S. Song , G. Kang , S. U. Ryu , T. Park , Adv. Energy Mater. 2018, 8, 1702235;

[21] L. Meng , C. Sun , R. Wang , W. Huang , Z. Zhao , P. Sun , T. Huang , J. Xue , J.‐W. Lee , C. Zhu , J. Am. Chem. Soc. 2018, 140, 17255. 30449094 10.1021/jacs.8b10520

[22] a) H.‐S. Kim , I.‐H. Jang , N. Ahn , M. Choi , A. Guerrero , J. Bisquert , N.‐G. Park , J. Phys. Chem. Lett. 2015, 6, 4633; 26551249 10.1021/acs.jpclett.5b02273

[23] T. Lemercier , L. Perrin , E. Planès , S. Berson , L. Flandin , Energies 2020, 13, 3794.

[24] a) D. P. Colombo Jr , K. A. Roussel , J. Saeh , D. E. Skinner , J. J. Cavaleri , R. M. Bowman , Chem. Phys. Lett. 1995, 232, 207;

[25] G. J. Hedley , A. Ruseckas , I. D. Samuel , Chem. Rev. 2017, 117, 796. 27951633 10.1021/acs.chemrev.6b00215 PMC5269644

[26] T. M. Clarke , J. R. Durrant , Chem. Rev. 2010, 110, 6736. 20063869 10.1021/cr900271s

[27] a) I. Campbell , T. Hagler , D. Smith , J. Ferraris , Phys. Rev. Lett. 1996, 76, 1900; 10060549 10.1103/PhysRevLett.76.1900

[28] N. S. Sariciftci , L. Smilowitz , A. J. Heeger , F. Wudl , Science 1992, 258, 1474. 17755110 10.1126/science.258.5087.1474

[29] C. W. Tang , Appl. Phys. Lett. 1986, 48, 183.

[30] a) M. Hiramoto , H. Fujiwara , M. Yokoyama , Appl. Phys. Lett. 1991, 58, 1062;

[31] a) B. Kippelen , J.‐L. Brédas , Energy Environ. Sci. 2009, 2, 251;

[32] A. Kojima , K. Teshima , Y. Shirai , T. Miyasaka , J. Am. Chem. Soc. 2009, 131, 6050. 19366264 10.1021/ja809598r

[33] Best Research‐Cell Efficiencies, https://www.nrel.gov/pv/assets/pdfs/best-research-cell-efficiencies.20190802.pdf. Aaccessed August, 2019.

[34] a) W. Shockley , H. J. Queisser , J. Appl. Phys. 1961, 32, 510;

[35] a) I. Mora‐Seró , Joule 2018, 2, 585;

[36] a) W. Tress , N. Marinova , O. Inganäs , M. K. Nazeeruddin , S. M. Zakeeruddin , M. Graetzel , Adv. Energy Mater. 2015, 5, 1400812;

[37] a) N. Marinova , W. Tress , R. Humphry‐Baker , M. I. Dar , V. Bojinov , S. M. Zakeeruddin , M. K. Nazeeruddin , M. Grätzel , ACS Nano 2015, 9, 4200; 25769194 10.1021/acsnano.5b00447

[38] M. Stolterfoht , V. M. Le Corre , M. Feuerstein , P. Caprioglio , L. J. A. Koster , D. Neher , ACS Energy Lett. 2019, 4, 2887.

[39] R. Saive , IEEE J. Photovoltaics 2019, 9, 1477.

[40] a) X. Sun , J. Xu , L. Xiao , J. Chen , B. Zhang , J. Yao , S. Dai , Int. J. Photoenergy 2017, 2017, 4935265;

[41] A. G. Martin , E. Keith , H. Yoshihiro , W. Wilhelm , D. D. Ewan , Prog. Photovoltaics 2012, 20, 12.

[42] W. Qarony , M. I. Hossain , A. Salleo , D. Knipp , Y. H. Tsang , Mater. Today Energy 2019, 11, 106.

[43] A. Tamang , A. Hongsingthong , P. Sichanugrist , V. Jovanov , H. Gebrewold , M. Konagai , D. Knipp , Sol. Energy Mater. Sol. Cells 2016, 144, 300.

[44] a) M. M. Lee , J. Teuscher , T. Miyasaka , T. N. Murakami , H. J. Snaith , Science 2012, 338, 643; 23042296 10.1126/science.1228604

[45] M. Liu , M. B. Johnston , H. J. Snaith , Nature 2013, 501, 395. 24025775 10.1038/nature12509

[46] a) Q. Ye , Y. Zhao , S. Mu , F. Ma , F. Gao , Z. Chu , Z. Yin , P. Gao , X. Zhang , J. You , Adv. Mater. 2019, 31, 1905143; 10.1002/adma.201905143 31631443

[47] Z. Chen , J. J. Wang , Y. Ren , C. Yu , K. Shum , Appl. Phys. Lett. 2012, 101, 093901.

[48] M. H. Kumar , S. Dharani , W. L. Leong , P. P. Boix , R. R. Prabhakar , T. Baikie , C. Shi , H. Ding , R. Ramesh , M. Asta , Adv. Mater. 2014, 26, 7122. 25212785 10.1002/adma.201401991

[49] Y. Rong , Y. Ming , W. Ji , D. Li , A. Mei , Y. Hu , H. Han , J. Phys. Chem. Lett. 2018, 9, 2707. 29738259 10.1021/acs.jpclett.8b00912

[50] M. M. Tavakoli , L. Gu , Y. Gao , C. Reckmeier , J. He , A. L. Rogach , Y. Yao , Z. Fan , Sci. Rep. 2015, 5, 14083. 26392200 10.1038/srep14083 PMC4585726

[51] M. R. Leyden , L. K. Ono , S. R. Raga , Y. Kato , S. Wang , Y. Qi , J. Mater. Chem. A 2014, 2, 18742.

[52] a) J. H. Heo , D. H. Song , S. H. Im , Adv. Mater. 2014, 26, 8179; 25348285 10.1002/adma.201403140

[53] A. Agresti , S. Pescetelli , A. L. Palma , A. E. Del Rio Castillo , D. Konios , G. Kakavelakis , S. Razza , L. Cinà , E. Kymakis , F. Bonaccorso , ACS Energy Lett. 2017, 2, 279.

[54] J. Seo , S. Park , Y. C. Kim , N. J. Jeon , J. H. Noh , S. C. Yoon , S. I. Seok , Energy Environ. Sci. 2014, 7, 2642.

[55] E. H. Jung , N. J. Jeon , E. Y. Park , C. S. Moon , T. J. Shin , T.‐Y. Yang , J. H. Noh , J. Seo , Nature 2019, 567, 511. 30918371 10.1038/s41586-019-1036-3

[56] L. Qiu , S. He , L. K. Ono , S. Liu , Y. Qi , ACS Energy Lett. 2019, 4, 2147.

[57] Y. Deng , X. Zheng , Y. Bai , Q. Wang , J. Zhao , J. Huang , Nat. Energy 2018, 3, 560.

[58] a) Y. Deng , E. Peng , Y. Shao , Z. Xiao , Q. Dong , J. Huang , Energy Environ. Sci. 2015, 8, 1544;

[59] S. Razza , F. Di Giacomo , F. Matteocci , L. CinÓ , A. L. Palma , S. Casaluci , P. Cameron , A. D'epifanio , S. Licoccia , A. Reale , J. Power Sources 2015, 277, 286.

[60] a) Y. Galagan , F. Di Giacomo , H. Gorter , G. Kirchner , I. de Vries , R. Andriessen , P. Groen , Adv. Energy Mater. 2018, 8, 1801935;

[61] F. Di Giacomo , S. Shanmugam , H. Fledderus , B. J. Bruijnaers , W. J. Verhees , M. S. Dorenkamper , S. C. Veenstra , W. Qiu , R. Gehlhaar , T. Merckx , Sol. Energy Mater. Sol. Cells 2018, 181, 53.

[62] J. H. Heo , M. H. Lee , M. H. Jang , S. H. Im , J. Mater. Chem. A 2016, 4, 17636.

[63] D.‐K. Lee , D.‐N. Jeong , T. K. Ahn , N.‐G. Park , ACS Energy Lett. 2019, 4, 2393.

[64] H. Chen , F. Ye , W. Tang , J. He , M. Yin , Y. Wang , F. Xie , E. Bi , X. Yang , M. Grätzel , Nature 2017, 550, 92. 28869967 10.1038/nature23877

[65] M. R. Leyden , Y. Jiang , Y. Qi , J. Mater. Chem. A 2016, 4, 13125.

[66] F. Sahli , J. Werner , B. A. Kamino , M. Bräuninger , R. Monnard , B. Paviet‐Salomon , L. Barraud , L. Ding , J. J. D. Leon , D. Sacchetto , Nat. Mater. 2018, 17, 820. 29891887 10.1038/s41563-018-0115-4

[67] S. Öz , J. Burschka , E. Jung , R. Bhattacharjee , T. Fischer , A. Mettenbörger , H. Wang , S. Mathur , Nano Energy 2018, 51, 632.

[68] a) I. Chung , J.‐H. Song , J. Im , J. Androulakis , C. D. Malliakas , H. Li , A. J. Freeman , J. T. Kenney , M. G. Kanatzidis , J. Am. Chem. Soc. 2012, 134, 8579; 22578072 10.1021/ja301539s

[69] a) W. Xiang , W. Tress , Adv. Mater. 2019, 31, 1902851; 10.1002/adma.201902851 31478275

[70] N. K. Noel , S. D. Stranks , A. Abate , C. Wehrenfennig , S. Guarnera , A.‐A. Haghighirad , A. Sadhanala , G. E. Eperon , S. K. Pathak , M. B. Johnston , Energy Environ. Sci. 2014, 7, 3061.

[71] F. Hao , C. C. Stoumpos , D. H. Cao , R. P. Chang , M. G. Kanatzidis , Nat. Photonics 2014, 8, 489.

[72] W. Ke , C. C. Stoumpos , I. Spanopoulos , L. Mao , M. Chen , M. R. Wasielewski , M. G. Kanatzidis , J. Am. Chem. Soc. 2017, 139, 14800. 28953381 10.1021/jacs.7b09018

[73] a) S. J. Lee , S. S. Shin , Y. C. Kim , D. Kim , T. K. Ahn , J. H. Noh , J. Seo , S. I. Seok , J. Am. Chem. Soc. 2016, 138, 3974; 26960020 10.1021/jacs.6b00142

[74] a) W. Ke , C. C. Stoumpos , I. Spanopoulos , M. Chen , M. R. Wasielewski , M. G. Kanatzidis , ACS Energy Lett. 2018, 3, 1470;

[75] Z. Zhu , C. C. Chueh , N. Li , C. Mao , A. K. Y. Jen , Adv. Mater. 2018, 30, 1703800. 10.1002/adma.201703800 29250846

[76] Q. Tai , X. Guo , G. Tang , P. You , T. W. Ng , D. Shen , J. Cao , C. K. Liu , N. Wang , Y. Zhu , Angew. Chem., Int. Ed. 2019, 58, 806. 10.1002/anie.201811539 30499609

[77] K. Chen , P. Wu , W. Yang , R. Su , D. Luo , X. Yang , Y. Tu , R. Zhu , Q. Gong , Nano Energy 2018, 49, 411.

[78] W. Ke , P. Priyanka , S. Vegiraju , C. C. Stoumpos , I. Spanopoulos , C. M. M. Soe , T. J. Marks , M.‐C. Chen , M. G. Kanatzidis , J. Am. Chem. Soc. 2018, 140, 388. 29211458 10.1021/jacs.7b10898

[79] E. Jokar , C. H. Chien , C. M. Tsai , A. Fathi , E. W. G. Diau , Adv. Mater. 2019, 31, 1804835. 10.1002/adma.201804835 30411826

[80] a) Z. Yi , N. H. Ladi , X. Shai , H. Li , Y. Shen , M. Wang , Nanoscale Adv. 2019, 1, 1276; 10.1039/c8na00416a PMC9418224 36132615

[81] K. Marshall , M. Walker , R. Walton , R. Hatton , Nat. Energy 2016, 1, 16178.

[82] T.‐B. Song , T. Yokoyama , S. Aramaki , M. G. Kanatzidis , ACS Energy Lett. 2017, 2, 897.

[83] H.‐D. Um , K.‐H. Choi , I. Hwang , S.‐H. Kim , K. Seo , S.‐Y. Lee , Energy Environ. Sci. 2017, 10, 931.

[84] a) M. A. Mahmoudzadeh , A. R. Usgaocar , J. Giorgio , D. L. Officer , G. G. Wallace , J. D. Madden , J. Mater. Chem. A 2016, 4, 3446;

[85] G.‐M. Weng , J. Kong , H. Wang , C. Karpovich , J. Lipton , F. Antonio , Z. S. Fishman , H. Wang , W. Yuan , A. D. Taylor , Energy Storage Mater. 2020, 24, 557.

[86] A. Gurung , K. Chen , R. Khan , S. S. Abdulkarim , G. Varnekar , R. Pathak , R. Naderi , Q. Qiao , Adv. Energy Mater. 2017, 7, 1602105.

[87] L.‐c. Kin , Z. Liu , O. Astakhov , S. N. Agbo , H. Tempel , S. Yu , H. Kungl , R. Eichel , U. Rau , T. Kirchartz , ACS Appl. Energy Mater. 2019, 3, 431.

[88] A. Gurung , K. M. Reza , S. Mabrouk , B. Bahrami , R. Pathak , B. S. Lamsal , S. I. Rahman , N. Ghimire , R. S. Bobba , K. Chen , J. Pokharel , A. Baniya , M. A. R. Laskar , M. Liang , W. Zhang , W.‐H. Zhang , S. Yang , K. Xu , Q. Qiao , Adv. Funct. Mater. 2020, 30, 2001865.

[89] S. Ahmad , C. George , D. J. Beesley , J. J. Baumberg , M. De Volder , Nano Lett. 2018, 18, 1856. 29425044 10.1021/acs.nanolett.7b05153

[90] a) B. D. Boruah , A. Mathieson , B. Wen , S. Feldmann , W. M. Dose , M. De Volder , Energy Environ. Sci. 2020, 13, 2414;

[91] B. D. Boruah , Energy Storage Mater. 2020, 34, 53.

[92] C. Li , S. Cong , Z. Tian , Y. Song , L. Yu , C. Lu , Y. Shao , J. Li , G. Zou , M. H. Rümmeli , Nano Energy 2019, 60, 247.

[93] X. Xu , S. Li , H. Zhang , Y. Shen , S. M. Zakeeruddin , M. Graetzel , Y.‐B. Cheng , M. Wang , ACS Nano 2015, 9, 1782. 25611128 10.1021/nn506651m

[94] P. Du , X. Hu , C. Yi , H. C. Liu , P. Liu , H. L. Zhang , X. Gong , Adv. Funct. Mater. 2015, 25, 2420.

[95] R. Liu , C. Liu , S. Fan , J. Mater. Chem. A 2017, 5, 23078.

[96] Z. Liu , Y. Zhong , B. Sun , X. Liu , J. Han , T. Shi , Z. Tang , G. Liao , ACS Appl. Mater. Interfaces 2017, 9, 22361. 28614655 10.1021/acsami.7b01471

[97] H. Sun , Y. Jiang , L. Qiu , X. You , J. Yang , X. Fu , P. Chen , G. Guan , Z. Yang , X. Sun , J. Mater. Chem. A 2015, 3, 14977.

[98] C. Li , M. M. Islam , J. Moore , J. Sleppy , C. Morrison , K. Konstantinov , S. X. Dou , C. Renduchintala , J. Thomas , Nat. Commun. 2016, 7, 13319. 27834367 10.1038/ncomms13319 PMC5114596

[99] J. Xu , Z. Ku , Y. Zhang , D. Chao , H. J. Fan , Adv. Mater. Technol. 2016, 1, 1600074.

[100] F. Zhou , Z. Ren , Y. Zhao , X. Shen , A. Wang , Y. Y. Li , C. Surya , Y. Chai , ACS Nano 2016, 10, 5900. 27159013 10.1021/acsnano.6b01202

[101] J. Duan , T. Hu , Y. Zhao , B. He , Q. Tang , Angew. Chem., Int. Ed. 2018, 57, 5746. 10.1002/anie.201801837 29603834

[102] F. Giraud , Z. M. Salameh , IEEE Trans. Energy Convers. 2001, 16, 1.

[103] H. Chen , H. Xu , S. Wang , T. Huang , J. Xi , S. Cai , F. Guo , Z. Xu , W. Gao , C. Gao , Sci. Adv. 2017, 3, eaao7233. 29255803 10.1126/sciadv.aao7233 PMC5733111

[104] Y. Hu , Y. Bai , B. Luo , S. Wang , H. Hu , P. Chen , M. Lyu , J. Shapter , A. Rowan , L. Wang , Adv. Energy Mater. 2019, 9, 1900872.

[105] X. Xia , Z. Ku , D. Zhou , Y. Zhong , Y. Zhang , Y. Wang , M. J. Huang , J. Tu , H. J. Fan , Mater. Horiz. 2016, 3, 588.

[106] M. Batmunkh , Y. L. Zhong , H. Zhao , Adv. Mater. 2020, 32, 2000631. 10.1002/adma.202000631 32578271

[107] P. P. Boix , K. Nonomura , N. Mathews , S. G. Mhaisalkar , Mater. Today 2014, 17, 16.

[108] Y. Zhou , X. Yin , Q. Zhang , N. Wang , A. Yamamoto , K. Koumoto , H. Shen , H. Lin , Mater. Today Energy 2019, 12, 363.

[109] a) T. J. Hsueh , J. M. Shieh , Y. M. Yeh , Prog. Photovoltaics 2015, 23, 507;

[110] a) M. Salado , M. Andresini , P. Huang , M. T. Khan , F. Ciriaco , S. Kazim , S. Ahmad , Adv. Funct. Mater. 2020, 30, 1910561;

[111] a) B. Sharma , S. Singh , K. Hossain , S. Mallick , P. Bhargava , D. Kabra , Sol. Energy 2020, 211, 1084;

[112] a) L. Gao , F. Zhang , X. Chen , C. Xiao , B. W. Larson , S. P. Dunfield , J. J. Berry , K. Zhu , Angew. Chem., Int. Ed. 2019, 58, 11737; 10.1002/anie.201905690 31218795

[113] a) W. Hui , Y. Yang , Q. Xu , H. Gu , S. Feng , Z. Su , M. Zhang , J. Wang , X. Li , J. Fang , Adv. Mater. 2020, 32, 1906374; 10.1002/adma.201906374 31799762

[114] a) S. Akin , N. Arora , S. M. Zakeeruddin , M. Graetzel , R. H. Friend , M. I. Dar , Adv. Energy Mater. 2020, 10, 1903090;

[115] a) F. Qiao , Y. Xie , Z. Weng , H. Chu , J. Energy Chem. 2020, 50, 230;

[116] a) B. Han , D. Xu , S. S. Chi , D. He , Z. Zhang , L. Du , M. Gu , C. Wang , H. Meng , K. Xu , Adv. Mater. 2020, 32, 2004793; 10.1002/adma.202004793 32930460

[117] a) S. Wu , Y. Chen , T. Jiao , J. Zhou , J. Cheng , B. Liu , S. Yang , K. Zhang , W. Zhang , Adv. Energy Mater. 2019, 9, 1902915;

[118] L. C. Kin , Z. Liu , O. Astakhov , S. N. Agbo , T. Merdzhanova , Mater. Today Energy 2019, 13, 22.

[119] Y. Hu , F. Bai , X. Liu , Q. Ji , X. Miao , T. Qiu , S. Zhang , ACS Energy Lett. 2017, 2, 2219.

[120] Q. Zeng , X. Zhang , X. Feng , S. Lu , Z. Chen , X. Yong , S. A. Redfern , H. Wei , H. Wang , H. Shen , Adv. Mater. 2018, 30, 1705393. 10.1002/adma.201705393 29333763

[121] J. Duan , Y. Zhao , B. He , Q. Tang , Angew. Chem. 2018, 130, 3849.

[122] D. H. Kim , J. H. Heo , S. H. Im , ACS Appl. Mater. Interfaces 2019, 11, 19123. 31070346 10.1021/acsami.9b03413

[123] Q. Wang , X. Zheng , Y. Deng , J. Zhao , Z. Chen , J. Huang , Joule 2017, 1, 371.

[124] H. Bian , Q. Wang , S. Yang , C. Yan , H. Wang , L. Liang , Z. Jin , G. Wang , S. F. Liu , J. Mater. Chem. A 2019, 7, 5740.

[125] Y. Wang , T. Zhang , M. Kan , Y. Zhao , J. Am. Chem. Soc. 2018, 140, 12345. 30247030 10.1021/jacs.8b07927

[126] C. Dong , X. Han , W. Li , Q. Qiu , J. Wang , Nano Energy 2019, 59, 553.

[127] W. Xiang , Z. Wang , D. J. Kubicki , W. Tress , J. Luo , D. Prochowicz , S. Akin , L. Emsley , J. Zhou , G. Dietler , Joule 2019, 3, 205.

[128] L. A. Frolova , Q. Chang , S. Y. Luchkin , D. Zhao , A. F. Akbulatov , N. N. Dremova , A. V. Ivanov , E. E. Chia , K. J. Stevenson , P. A. Troshin , J. Mater. Chem. C 2019, 7, 5314.

[129] B. Abdollahi Nejand , V. Ahmadi , H. R. Shahverdi , ACS Appl. Mater. Interfaces 2015, 7, 21807. 26402149 10.1021/acsami.5b05477

[130] M. Saliba , T. Matsui , K. Domanski , J.‐Y. Seo , A. Ummadisingu , S. M. Zakeeruddin , J.‐P. Correa‐Baena , W. R. Tress , A. Abate , A. Hagfeldt , Science 2016, 354, 206. 27708053 10.1126/science.aah5557

[131] H. Tsai , W. Nie , J.‐C. Blancon , C. C. Stoumpos , R. Asadpour , B. Harutyunyan , A. J. Neukirch , R. Verduzco , J. J. Crochet , S. Tretiak , Nature 2016, 536, 312. 27383783 10.1038/nature18306

[132] Z. Wang , Q. Lin , F. P. Chmiel , N. Sakai , L. M. Herz , H. J. Snaith , Nat. Energy 2017, 2, 17135.

[133] N. Arora , M. I. Dar , A. Hinderhofer , N. Pellet , F. Schreiber , S. M. Zakeeruddin , M. Grätzel , Science 2017, 358, 768. 28971968 10.1126/science.aam5655

[134] B. Suarez , V. Gonzalez‐Pedro , T. S. Ripolles , R. S. Sanchez , L. Otero , I. Mora‐Sero , J. Phys. Chem. Lett. 2014, 5, 1628. 26270357 10.1021/jz5006797

[135] M. Higgins , F. Ely , R. C. Nome , R. A. Nome , D. P. Dos Santos , H. Choi , S. Nam , M. Quevedo‐Lopez , J. Appl. Phys. 2018, 124, 065306.

[136] H. Zheng , G. Liu , C. Zhang , L. Zhu , A. Alsaedi , T. Hayat , X. Pan , S. Dai , Sol. Energy 2018, 159, 914.

[137] L. Calio , M. Salado , S. Kazim , S. Ahmad , Joule 2018, 2, 1800.

[138] J.‐Y. Seo , H.‐S. Kim , S. Akin , M. Stojanovic , E. Simon , M. Fleischer , A. Hagfeldt , S. M. Zakeeruddin , M. Grätzel , Energy Environ. Sci. 2018, 11, 2985.

[139] S. Bai , P. Da , C. Li , Z. Wang , Z. Yuan , F. Fu , M. Kawecki , X. Liu , N. Sakai , J. T.‐W. Wang , Nature 2019, 571, 245. 31292555 10.1038/s41586-019-1357-2

[140] S. Wu , Z. Li , M.‐Q. Li , Y. Diao , F. Lin , T. Liu , J. Zhang , P. Tieu , W. Gao , F. Qi , Nat. Nanotechnol. 2020, 15, 934. 32958933 10.1038/s41565-020-0765-7

[141] J. H. Heo , H. J. Han , D. Kim , T. K. Ahn , S. H. Im , Energy Environ. Sci. 2015, 8, 1602.

[142] C.‐H. Chiang , J.‐W. Lin , C.‐G. Wu , J. Mater. Chem. A 2016, 4, 13525.

[143] T. Bu , X. Liu , J. Li , W. Huang , Z. Wu , F. Huang , Y.‐B. Cheng , J. Zhong , Sol. RRL 2020, 4, 1900263.

[144] G. S. Han , J. Kim , S. Bae , S. Han , Y. J. Kim , O. Y. Gong , P. Lee , M. J. Ko , H. S. Jung , ACS Energy Lett. 2019, 4, 1845.

[145] S. Pescetelli , A. Agresti , S. Razza , L. A. Castriotta , A. Di Carlo , Int. Symp. Adv. Electr. Commun. Technol. (ISAECT) 2019.

[146] Y. Deng , C. H. Van Brackle , X. Dai , J. Zhao , B. Chen , J. Huang , Sci. Adv. 2019, 5, eaax7537. 31840067 10.1126/sciadv.aax7537 PMC6897546

[147] S. Tian , J. Li , S. Li , T. Bu , Y. Mo , S. Wang , W. Li , F. Huang , Sol. Energy 2019, 183, 386.

[148] L. Luo , Y. Zhang , N. Chai , X. Deng , J. Zhong , F. Huang , Y. Peng , Z. Ku , Y.‐B. Cheng , J. Mater. Chem. A 2018, 6, 21143.

[149] L. Qiu , S. He , Y. Jiang , D.‐Y. Son , L. K. Ono , Z. Liu , T. Kim , T. Bouloumis , S. Kazaoui , Y. Qi , J. Mater. Chem. A 2019, 7, 6920.

[150] A. Gurung , K. M. Reza , S. Mabrouk , B. Bahrami , R. Pathak , B. S. Lamsal , S. I. Rahman , N. Ghimire , R. S. Bobba , K. Chen , Adv. Funct. Mater. 2020, 30, 2001865.

